# MiR-22 modulates brown adipocyte thermogenesis by synergistically activating the glycolytic and mTORC1 signaling pathways

**DOI:** 10.7150/thno.50900

**Published:** 2021-01-25

**Authors:** Pengbo Lou, Xueyun Bi, Yuhua Tian, Guilin Li, Qianqian Kang, Cong Lv, Yongli Song, Jiuzhi Xu, Xiaole Sheng, Xu Yang, Ruiqi Liu, Qingyong Meng, Fazheng Ren, Maksim V. Plikus, Bin Liang, Bing Zhang, Huiyuan Guo, Zhengquan Yu

**Affiliations:** 1 State Key Laboratories for Agrobiotechnology and Key Laboratory of Precision Nutrition and Food Quality, Ministry of Education, Department of Nutrition and Health, College of Biological Sciences, China Agricultural University, Beijing, China, 100193.; 2 Key Laboratory of Precision Nutrition and Food Quality, Ministry of Education, Department of Nutrition and Health, College of Food Sciences and nutritional engineering, China Agricultural University, Beijing, China, 100083.; 3 Department of Developmental and Cell Biology, Sue and Bill Gross Stem Cell Research, Center for Complex Biological Systems, University of California, Irvine, Irvine, CA 92697, USA.; 4 Center for Life Sciences, School of Life Sciences, Yunnan University, Kunming, Yunnan, China, 650091.; 5 College of Veterinary Medicine, China Agricultural University, Beijing, China.

**Keywords:** miR-22, BAT, thermogenesis, glycolysis, mTORC1

## Abstract

**Background:** Brown adipose tissue (BAT) dissipates chemical energy as heat and has the potential to be a protective strategy to prevent obesity. microRNAs (miRNAs) are emerging as important posttranscriptional factors affecting the thermogenic function of BAT. However, the regulatory mechanism underlying miRNA-mediated energy metabolism in BAT is not fully understood. Here, we explored the roles of miR-22 in BAT thermogenesis and energy metabolism.

**Methods:** Using global and conditional knockout mice as *in vivo* models and primary brown adipocytes as an *in vitro* system, we investigated the function of miR-22 in BAT thermogenesis *in vivo* and *in vitro*.

**Results:** miR-22 expression was upregulated in BAT in response to cold exposure and during brown preadipocyte differentiation. Both global and conditional knockout mice displayed BAT whitening, impaired cold tolerance, and decreased BAT thermogenesis. Moreover, we found that miR-22 deficiency impaired BAT glycolytic capacity, which is critical for thermogenesis. The mechanistic results revealed that miR-22 activated the mTORC1 signaling pathway by directly suppressing Tsc1 and concomitantly directly suppressing Hif1an, an inhibitor of Hif1α, which promotes glycolysis and maintains thermogenesis.

**Conclusions:** Our findings identify miR-22 as a critical regulator in the control of thermogenesis in BAT and as a potential therapeutic target for human metabolic disorders.

## Introduction

The incidence of obesity has increased in recent decades, and obesity leads to numerous comorbidities, including cardiovascular disease, metabolic syndrome, and type 2 diabetes 1. In addition to reducing calorie intake, increasing energy expenditure (EE) is also an alternative strategy to prevent obesity. Brown adipose tissue (BAT) is a specialized tissue that specifically expresses uncoupling protein 1 (Ucp1), which uncouples oxidative respiration from ATP synthesis to generate heat 2. This core function of BAT allows rodents to maintain their body temperature in cold environments 3. Human infants have copious BAT deposits, but humans lose prominent BAT depots after becoming adults. Notably, increasing evidence indicates that adult humans also possess Ucp1-positive BAT 4, 5, which contributes to metabolic homeostasis. Such findings have uncovered a viable therapeutic strategy, where these cells are exploited to treat obesity and diabetes 6, 7. Thus, understanding the regulatory mechanism that drives BAT thermogenic activity now has clinical implications.

Upon cold exposure, BAT is activated. During this process, Ucp1 dissipates the proton gradient across the inner mitochondrial membrane and then uncouples oxidative respiration from ATP synthesis to generate heat. To compensate for the decrease in ATP, glycolytic activity is increased in BAT when mice or humans are subjected to cold stimulation 8. Prevention of glycolysis in mouse BAT severely impairs nonshivering thermogenesis (NST); consequently, mice in which glycolysis is inhibited are hypothermic, and display increased sensitivity to cold 9, 10, suggesting the importance of glycolysis in BAT thermogenesis. However, the regulatory mechanism of glycolysis remains unclear.

The mammalian target of rapamycin (mTOR) signaling pathway, which is a central regulator of energy metabolism in cells, plays important roles in maintaining BAT thermogenesis 11, 12. In response to cold, mTORC1 activity is robustly increased in BAT. Loss of *Raptor* in adipocytes blocks cold-induced BAT mass expansion, reduces mitochondrial biogenesis, and severely impairs BAT oxidative metabolism 13. Inhibition of mTORC1 signaling via rapamycin renders mice cold intolerant, as it blocks the expression of thermogenic genes and the expansion of beige adipocytes in white adipose tissue (WAT) 14, 15. However, the molecular mechanism involved in mTOR activation upon cold exposure is still unclear.

MicroRNAs (miRNAs) are a class of short, noncoding RNAs that control multiple developmental and cellular processes by negatively regulating gene expression 16. Recently, increasing evidence has indicated that miRNAs are important regulators of thermogenic function in BAT 17. For example, it has been reported that miR-27 and miR-133 inhibit brown adipogenesis, while miR-32 and miR-455 drive BAT thermogenesis 18-21. miR-22 is of great interest, as it plays a critical role in regulating lipid metabolism in muscle, and both muscle and BAT are derived from the same progenitors that express the early muscle marker myogenic factor 5 (MYF5) and paired-box 7 (Pax7) 22, 23. miR-22 has also been suggested to be important in a variety of cardiac diseases 24-26. However, the function of miR-22 in BAT is completely unknown. Here, we utilized both global and conditional knockout mice models to demonstrate that cold-induced miR-22 drives thermogenic function in BAT by modulating glycolysis and thermogenic genes via the Hif1an/Hif1α and mTORC1 signaling pathways.

## Materials and methods

### Ethics statement

This study was performed in strict accordance with the recommendations in the Regulations of Beijing Laboratory Animal Management. The protocol was approved by the Institutional Animal Care and Use Committee of China Agricultural University (approval number: SKLAB-2015-04-03).

### Animals

The miR-22 KO mice, *Adipoq-Cre* and* Ucp1-Cre* transgenic mice were obtained from the Jackson Laboratory. The *miR-22^flox/flox^* mice were generated at the Shanghai Model Animal Center, and the second exon (337 bp) of miR-22 was targeted with flanking LoxP sites, resulting in the 2 LoxP loci. Experimental groups were formed according to the genotype and contained male mice that were similar in age. Single-housed mice were placed at 4 °C or 30 °C for cold exposure and thermoneutral experiments.

### miR-22 *in situ* hybridizations and quantitative real-time PCR (qRT-PCR)

For miR-22 *in situ* hybridizations, digoxigenin (DIG)-labeled locked nucleic acid (LNA) probes (Exiqon, Vedbaek, Denmark) were used following the manufacturer's protocol. Double DIG-labeled miR-22 and scrambled LNA probes (Exiqon) were hybridized at 55 °C. The *in situ* signals were detected by staining with an anti-DIG-AP antibody (Roche) and developed using BM purple substrate (Roche).

Total RNA was extracted by TRIzol (Invitrogen) according to the manufacturer's instructions. Each RNA sample was reverse transcribed with the M-MLV Reverse Transcriptase (Promega) using oligo (dT) primers. Real-time qPCR was performed using LightCycler 480 SYBR Green I Master Mix on a detection system (Roche). The primers used are located in Table S1.

### Cell culture and transfection

Stromal vascular fraction (SVF) cells were isolated from BAT as described previously 27 with minor modifications. Briefly, BAT was excised from newborn mice, minced and digested with collagenase type II collagenase (2 mg/mL) at 37 °C for 90 min, then filtered and centrifuged. Finally, SVF cells were resuspended in culture medium.

For differentiation assays, SVF cells were cultured to confluence and treated with induction medium containing 10% FBS, 0.5 mM isobutylmethylxanthine, 125 nM indomethacin, 2 μg/mL dexamethasone, 0.5 μM rosiglitazone, 5 μg/mL insulin, and 1 nM T3. After 2 days, the cells were incubated with maintenance medium containing 5 μg/mL insulin and 1 nM T3 for 2 days. Then, the cells were incubated in DMEM with 10% FBS for 2 days.

Differentiated primary brown adipocytes were transfected as reported 28. In brief, Lipofectamine RNAiMAX (9 μL/mL) and inhibitor/negative control (NC) or mimic/NC (50 nM) were diluted with Opti-MEM medium and mixed them (1:1). The mix was added to a plate (precoated with gelatin) and then incubated for 25 min. During incubation, digested differentiated primary brown adipocytes (day 4) were counted and resuspended in culture medium. The resuspended cells were added to plates and analyzed after 2 days. The sequences of siRNA or inhibitor used are listed in Table S2. The miR-22 lentiviral vector was generated by cloning 695 bp of mouse genomic DNA harboring the pri-miR-22 sequence into the pLV[Exp]-EGFP:T2A:Puro-EF1A vector.

### Cellular metabolism

Oxygen consumption rate (OCR) and extracellular acidification rate (ECAR) assays were performed and analyzed as reported 29, 30. In brief, SVF cells from BAT of miR-22 KO mice or wild-type (WT) mice were seeded in an XFe24 cell culture microplate (Agilent) and differentiated into brown adipocytes. Cells at day 6 of differentiation were equilibrated in Seahorse XF Base Medium (Agilent) for 1 H in a 37 °C incubator without CO_2_ before analysis. Then, the XFe24 plates were transferred to a Seahorse XFe24 analyzer, equilibrated for 15 min and subjected to 3 assay cycles (mixing for 3 min, waiting for 2 min and rate measurement for 2 min). A cell Mito Stress Kit was used for the measurement of OCR (oligomycin, 1 μM; carbonyl cyanide-4-(trifluoromethoxy) phenylhydrazone (FCCP), 2 μM; and rotenone/antimycin A, 0.5 μM), and a Glycolysis Stress Kit was used for the measurement of the ECAR (glucose, 10 mM; oligomycin, 1 μM; and 2-deoxyglucose (2-DG), 50 mM). After the assays were performed, total proteins were extracted from each well and quantified using a BCA protein quantification kit for normalization. The data were analyzed using Seahorse Wave Desktop Software (Agilent).

### Tissue O_2_ consumption and glycolysis

The OCR and ECAR in interscapular BAT were measured using a Seahorse XFe Extracellular Flux Analyzer (Agilent) in a 24-well plate. Adipose tissues (2 mg for BAT) were placed into XF24 Islet Capture Microplates and preincubated with assay medium for 1 H according to the manufacturer's instructions (Agilent).

### Hematoxylin and eosin (H&E), immunofluorescence, immunohistochemistry (IHC) and western blotting

H&E staining, immunofluorescence, IHC and western blotting were performed as we previously described 31. The BAT was rinsed with PBS, fixed in 4% PFA, paraffin-embedded, sectioned at 5 μm and stained with H&E. For immunohistochemical staining, antigen retrieval was performed by heating the slides to 95 °C for 20 min in 0.01 M citrate buffer (pH 6.0) in a microwave. IHC was performed using an Expose Kit (Abcam) according to the manufacturer's protocol. The antibodies used are listed in Table S3.

BAT or cells were prepared in lysis buffer (Beyotime Biotechnology, Shanghai, China) with 1% phenylmethylsulfonyl fluoride (PMSF). The protein concentration was measured using a BCA protein assay kit (Beyotime). Thirty micrograms of total protein was separated by 8%-10% SDS-PAGE under denaturing conditions, and the separated proteins were transferred to PVDF membranes (GE Healthcare). The membranes were blocked with 5% BSA for 1 H, incubated with the primary antibodies overnight at 4 °C, washed, and incubated with the appropriate horseradish-conjugated secondary antibody (1:5,000) for 1 H at room temperature.

### Indirect calorimetry core body temperature measurement

Oxygen consumption (VO_2_) and carbon dioxide production (VCO_2_) were measured for 24 H after 24 H of acclimation using an Oxylet System (Panlab, Barcelona, Spain) and the METABOLISM software suite. Mice were housed in single cages at 4 °C for a period of 0-6 H, and their core body temperatures were measured with a probe thermometer.

### Microarray analyses

For mRNA microarrays, total RNA was extracted from BAT samples isolated from WT and miR-22 KO mice (8 weeks old) using TRIzol Reagent (Invitrogen). Then, the RNA samples were submitted to the Beijing CapitalBio Corporation and analyzed on Agilent cDNA microarrays (Mouse (V2) Gene Expression Microarray). The data were analyzed using Significant Analysis of Microarray (SAM) software to identify the differentially expressed genes. Genes were considered to be significantly upregulated or downregulated at p < 0.05. We utilized the Database for Annotation, Visualization and Integrated Discovery (DAVID) Functional Annotation Bioinformatics Microarray Analysis software to analyze Gene Ontology (GO) terms based on the Kyoto Encyclopedia of Genes and Genomes (KEGG) pathway database.

### Luciferase reporter assays

Approximately 650 bp segments containing predicted miRNA target sites in the 3'UTRs of *Hif1an*, *Tsc1* were cloned into psiCHECK-2 vectors (Promega) between the *XhoI* and *NotI* sites immediately downstream of the Renilla luciferase gene. To generate reporters with mutant 3'UTRs, six nucleotides (GCAGCT) in the target sites were mutated to TAGATC.

Then, 293T cells were seeded in 24-well plates one day before transfection. Ten nanograms of each reporter construct was cotransfected with a miR-22 mimic or a NC at a final concentration of 50 nM into 293T cells using Lipofectamine 2000 according to the manufacturer's protocol (Invitrogen). The next day, the firefly and renilla luciferase activities were measured with a Dual-Glo Luciferase Assay System according to the manufacturer's instructions (Promega).

### Statistical analysis

Two-tailed Student's *t*-test and two-way ANOVA were used for statistical analysis. The data are reported as the mean ± SD unless specifically stated to be the mean ± SEM in the figure legends.

### Data availability

The microarray data have been submitted to the GEO repository GSE147835.

## Results

### miR-22 is highly expressed in BAT and upregulated upon cold exposure

**Figure 1:**
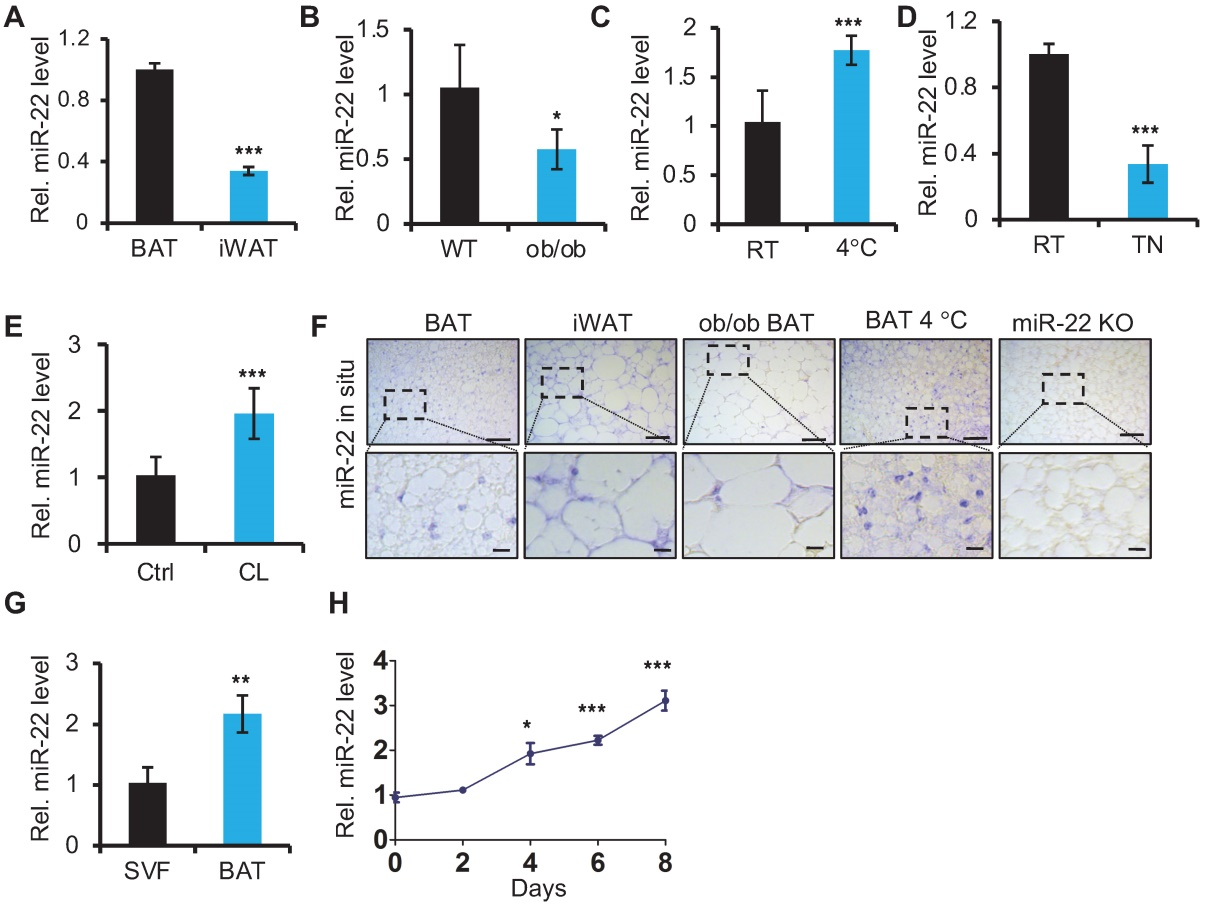
miR-22 is highly expressed in brown fat and is upregulated upon cold exposure or during differentiation. (A) qRT-PCR analysis for miR-22 in BAT and iWAT from 8-week-old mice. n = 3 biological replicates. (B) qRT-PCR analysis for miR-22 in BAT of WT and ob/ob mice. n = 3 biological replicates. (C) qRT-PCR analysis for miR-22 in BAT from mice at room temperature (RT) and after exposure to 4 °C for 24 H. n = 3 biological replicates. (D) qRT-PCR analysis for miR-22 in BAT from mice at RT and thermoneutrality (TN). (E) qRT-PCR analysis for miR-22 in BAT in response to CL (CL316,243) injection (1 mg/kg, 6 H). n = 3 biological replicates. (F) *In situ* hybridization for miR-22 at indicated conditions. Top panel, representative low-magnification images (scale bar: 50 µm); bottom panels, high-magnification images of the areas indicated by the dashed boxes in the top panel images (scale bar: 10 µm). miR-22 KO BAT was used as a negative control. (G) qRT-PCR analysis for miR-22 in SVF and mature adipocytes isolated from the BAT of 8-week-old WT mice. (H) qRT-PCR analysis for miR-22 in brown preadipocytes during differentiation. *P < 0.05, **P < 0.01, and ***P < 0.001 (two-tailed Student's *t*-test).

To begin elucidating the potential role of miR-22 in adipose tissue, we first examined the miR-22 expression levels in BAT. We found that the expression levels of miR-22 were higher in BAT than in inguinal WAT (iWAT) (Figure 1A). Its expression levels in BAT from ob/ob or db/db mice, in which BAT thermogenic function is impaired 32, were significantly lower than in WT mice (Figure 1B, Figure S1A). BAT thermogenesis is normally activated under cold stimulation and inactivated under thermoneutral conditions 2. The miR-22 levels in BAT exhibited significant upregulation upon cold exposure; in contrast, miR-22 was significantly downregulated under thermoneutral conditions (Figure 1C-D). Consistently, the miR-22 expression level was significantly upregulated in BAT or differentiated primary brown adipocyte adipocytes upon CL316, 243 treatment, which mimics cold stimuli (Figure 1E, Figure S1B). The findings were further confirmed by miR-22 *in situ* hybridization (Figure 1F, Figure S1C). Furthermore, we found that the miR-22 expression level was higher in mature BAT than that in the SVF (Figure 1G). In line with these results, the miR-22 level increased with time during adipogenesis with time (Figure 1H). Collectively, these findings indicated that miR-22 is highly expressed in mature BAT and responds to cold stimuli.

### miR-22 deficiency results in thermogenic dysfunction

**Figure 2:**
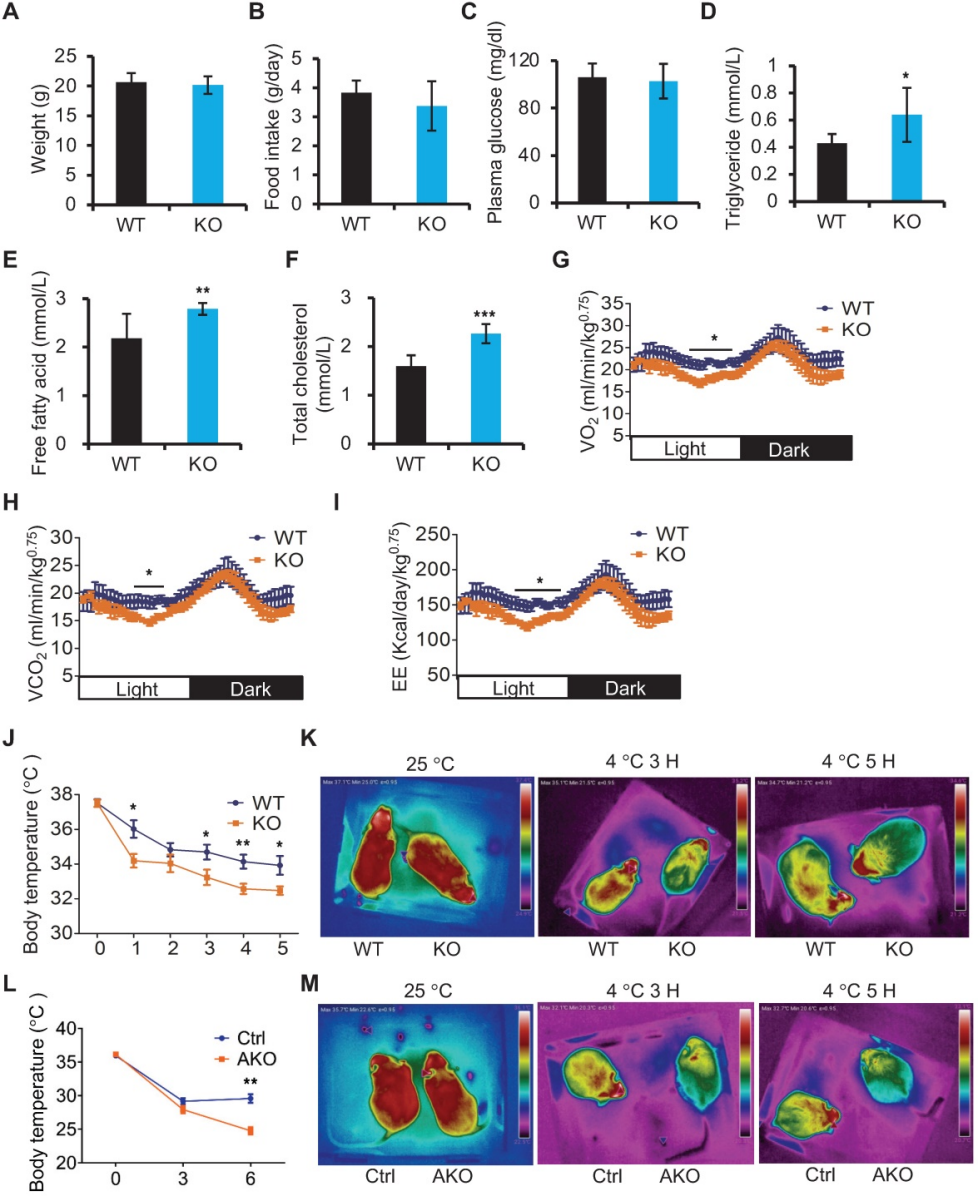
miR-22 is required for acute BAT-mediated thermogenesis. (A) Body weights of WT (n = 10) and KO (n = 10) male mice at the age of 12 weeks. (B) Food intake of WT (n = 10) and KO (n = 10) male mice at the age of 12 weeks. (C-F) The levels of Glucose (C), triglyceride (TAG) (D), free fatty acid (E), and total cholesterol (F) in the serum from WT (n = 8) and KO (n = 8) male mice at the age of 10 weeks under 16-hour fasted conditions. (G-I) Indirect calorimetry analysis of oxygen consumption (VO_2_) (G), exhaled carbon dioxide (VCO_2_) (H), and EE (I) in WT (n = 8) and KO (n = 8) male mice at the age of 10 weeks. (J) Quantification of core body temperatures of miR-22 KO mice (n = 8) and their littermate controls (n = 8) at different time points after exposure to 4 °C. The mice were treated with food and water deprivation after cold exposure. (K) Representative thermal images of miR-22 KO mice (n = 3) and their littermate controls (n = 3) at the indicated conditions. (L) Quantification of core body temperatures of miR-22 AKO mice (n = 10) and their littermate controls (n = 10) at different time points after exposure to 4 °C. The mice were treated with food and water deprivation after cold exposure. (M) Representative thermal images of miR-22 AKO mice (n = 3) and their littermate controls (n = 3) at the indicated conditions. *P < 0.05, **P < 0.01, and ***P < 0.001 (two-tailed Student's *t*-test). Data are represented as the means ± SEMs (G-I) and others as the means ± SDs. AKO: adipocyte-specific miR-22 knockout mice, *Adipoq-Cre*; *miR-22^f/f^*.

Next, we utilized miR-22 KO mice to study the *in vivo* role of miR-22 in BAT. Compared with WT mice, miR-22 KO mice fed on a normal chow diet did not show any noticeable differences in outward appearance. The body weights and food intakes were not significantly different between the miR-22 KO mice and their WT littermates (Figure 2A-B). With regard to serum metabolites, the levels of circulating plasma glucose were also normal in miR-22 KO mice (Figure 2C). However, the free fatty acid, triglyceride and total cholesterol levels were significantly higher in miR-22 KO mice than in littermate controls (Figure 2D-F). Accordingly, we also observed significant decreases in VO_2_, VCO_2_ and EE in miR-22 KO mice (Figure 2G-I). These findings indicate the occurrence of systemic metabolic dysfunction in miR-22 KO mice.

The primary function of BAT is to generate heat to defend against cold exposure 33. Considering the high levels of miR-22 in BAT, we examined body temperature in miR-22 KO mice subjected to acute cold exposure. The body temperatures of miR-22 KO mice dropped much faster than those of the littermate controls (Figure 2J). Thermographic imaging further revealed that the BAT surface temperature rapidly decreased in miR-22 KO mice upon acute cold exposure (Figure 2K), indicating defective adaptive thermogenesis in BAT. Taken together, these results suggest the potential importance of miR-22 in metabolic homeostasis and thermogenic function.

To test whether the phenotypes observed upon miR-22 loss originated in BAT, we examined the phenotypes using Adipoq-Cre-driven miR-22 conditional KO (AKO) mice in which miR-22 was specifically deleted in adipose tissues (Figure S2A-B). Interestingly, compared with WT littermate controls, AKO mice did not exhibit any significant changes in serum biochemical indices or metabolic cage parameters (Figure S2C-I), but significant alterations were observed in constitutive KO mice. It turns out that the metabolic changes found in KO mice could be caused by miR-22 knockout in tissues other than adipose tissues. However, similar to constitutive KO mice, the miR-22 AKO mice failed to maintain their core body temperatures or interscapular BAT temperatures upon acute cold exposure (Figure 2L-M).

To further delineate the specific function of miR-22 in BAT for cold-induced NST, we generated *Ucp1-Cre;miR-22^fl/fl^* (BKO) mice, in which miR-22 is specifically deleted in BAT (Figure S2J). The interscapular BAT temperature in miR-22 BKO mice dropped more rapidly upon acute cold exposure, compared to control mice (Figure S2K). These findings suggest that adipocyte-specific miR-22 is specifically important for maintaining body temperature but does not affect whole-body metabolic homeostasis under a normal chow diet.

### miR-22 deficiency results in BAT whitening

**Figure 3:**
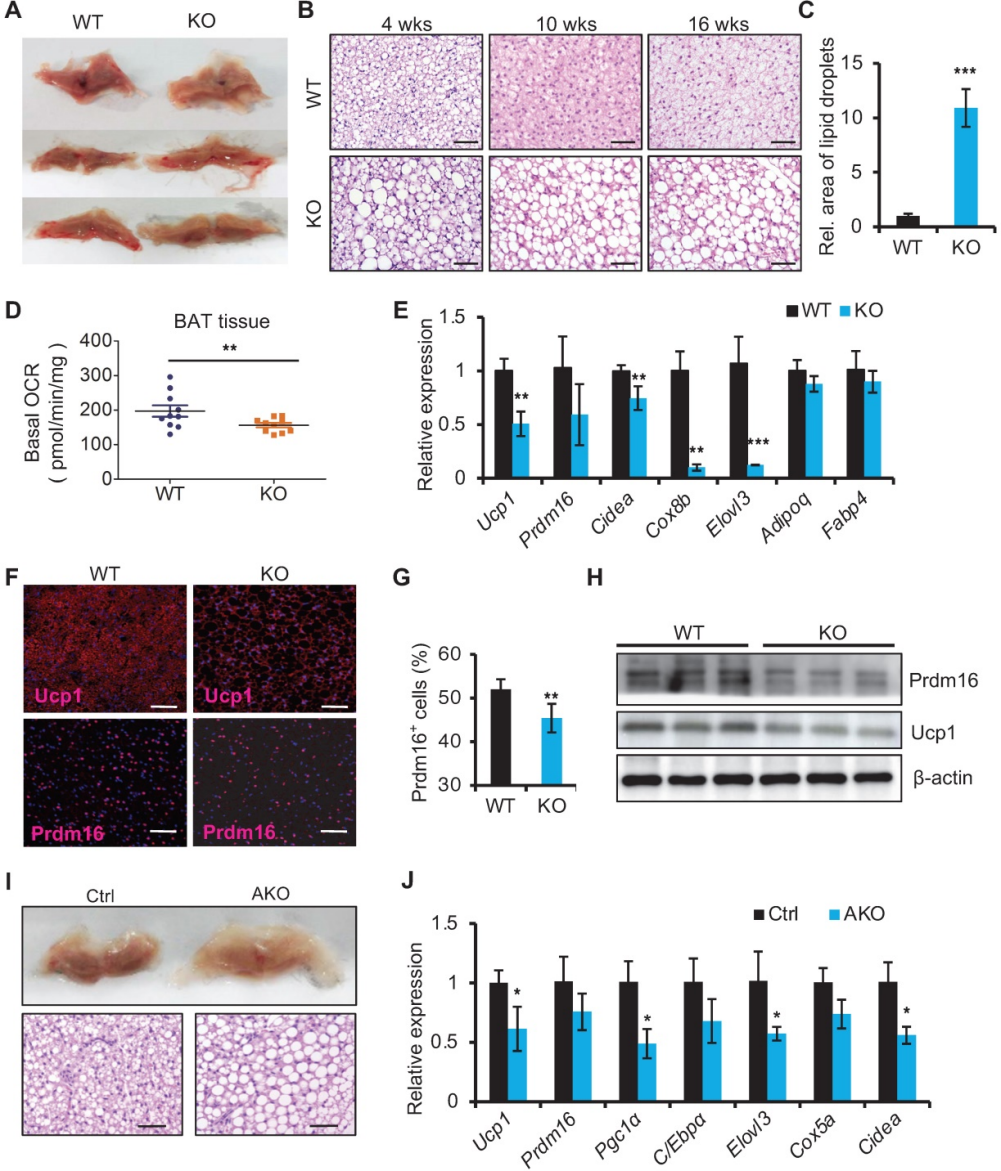
Whitening of BAT in miR-22 KO mice. (A) Macroscopic view of BAT miR-22 KO mice and their littermate controls at the age of 10 weeks. (B) Histological images of BAT from miR-22 KO mice and their littermate controls at the indicated time points. n = 5 for each timepoints. Scale bar: 50 µm. (C) Relative sizes of the lipid droplets from miR-22 KO mice and their littermate controls at the age of 10 weeks. n = 3 biological replicates. (D) O_2_ consumption of isolated BAT from WT and miR-22 KO mice. n = 10 biological replicates. (E) qRT-PCR analysis for BAT-selective and pan-adipocyte genes in BAT from miR-22 KO mice and their littermate controls. n = 3 biological replicates. (F) Immunofluorescence of Ucp1 and Prdm16 in BAT from miR-22 KO mice and their littermate controls. n = 3 biological replicates. Scale bar: 50 µm. (G) Quantification of Prdm16^+^ cells in Panel F. (H) Western blots for Ucp1 and Prdm16 in BAT from WT and miR-22 KO mice. β-actin was used as a loading control. (I) Macroscopic and histological images of control and miR-22 AKO mice BAT at the age of 10 weeks. n = 3 biological replicates. Scale bar: 50 µm. (J) qRT-PCR analysis for BAT-selective genes in BAT from control and miR-22 AKO mice at the age of 10 weeks. n = 3 biological replicates. *P < 0.05, **P < 0.01, and ***P < 0.001 (two-tailed Student's *t*-test).

We next examined the histology of BAT from miR-22 KO mice. On gross examination, BAT from miR-22 KO mice exhibited profound morphological whitening (Figure 3A). The whitening was accompanied by a switch from a multilocular to a unilocular morphology (Figure 3B) and an increase in lipid areas (Figure 3C). To test the BAT thermogenesis-related function of miR-22, we assessed NST by measuring BAT O_2_ consumption *in vitro*. The O_2_ consumption of BAT isolated from miR-22 KO mice was significantly lower than that of BAT isolated from littermate controls (Figure 3D). Consistently, BAT-selective genes were significantly downregulated in miR-22 KO mice (Figure 3E). The protein expression of Ucp1 and Prdm16 was also attenuated in miR-22 KO mice (Figure 3F-H). In line with these findings, the BAT from miR-22-AKO mice also exhibited profound morphological whitening, downregulation of BAT-selective genes and decreased O_2_ consumption (Figure 3I-J, Figure S3A-C). Furthermore, miR-22 BKO mice also exhibited the whitening of BAT and downregulation of BAT-selective genes, compared to control mice (Figure S3D-E), suggesting a specific role of miR-22 in BAT. Together, these *in vivo* findings demonstrate that miR-22 plays an important role in modulating thermogenic function of BAT.

### miR-22 is required for a brown adipocyte-specific thermogenic program in isolated BAT precursors

**Figure 4:**
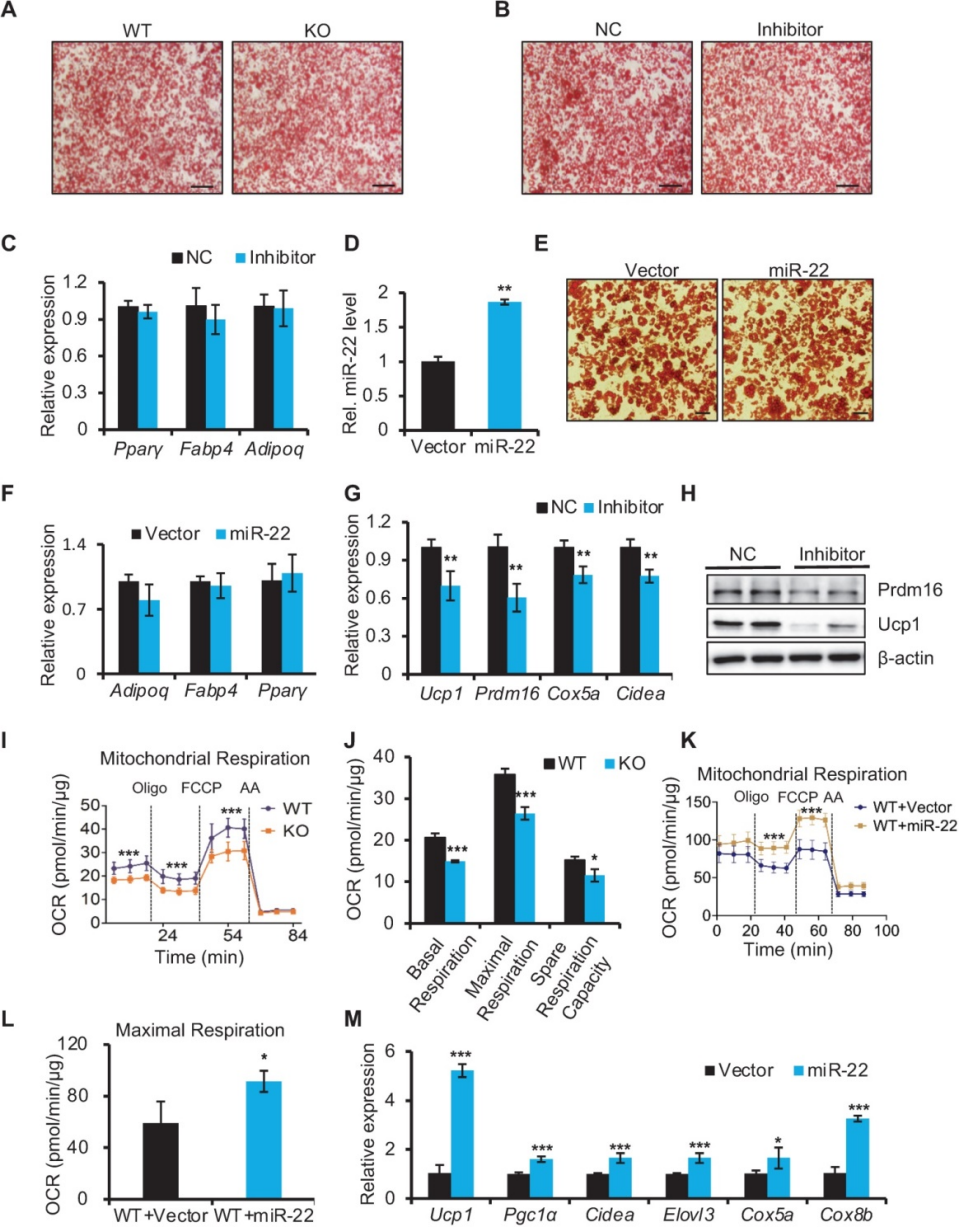
miR-22 is required for activation of the BAT-selective gene program *in vitro*. (A-B) Oil red O staining of differentiated primary brown adipocytes (day 6) from WT and miR-22 KO mice (A), or primary brown adipocytes treated with scramble RNA and miR-22 inhibitor (B). Scale bar: 200 µm. (C) qRT-PCR analysis for pan-adipocyte genes in differentiated primary brown adipocytes upon miR-22 inhibitor treatment. n = 3 technical replicates. (D) qRT-PCR analysis for miR-22 in primary brown adipocytes (differentiated for 6 days) upon miR-22 overexpression by lentiviral transduction. n = 4 technical replicates. (E) Oil red O staining of differentiated primary brown adipocytes (day 6) upon miR-22 overexpression. Scale bar: 50 µm. (F) qRT-PCR analysis for pan-adipocyte genes in primary brown adipocytes (differentiated for 6 days) upon miR-22 overexpression by lentiviral transduction. n = 4 technical replicates. (G) qRT-PCR analysis for BAT-selective genes in differentiated primary brown adipocytes upon miR-22 inhibitor treatment. n = 3 technical replicates. (H) Western blots for Ucp1 and Prdm16 in differentiated primary brown adipocytes upon miR-22 inhibitor treatment. β-actin was used as a loading control. (I-J) OCR in WT and miR-22 KO differentiated primary brown adipocytes (day 6). Oligomycin (oligo), FCCP and rotenone/antimycin A (AA) were added at the time points indicated by the dashed lines. (K-L) OCR in differentiated primary brown adipocytes (day 6) upon miR-22 overexpression. (M) qRT-PCR analysis for BAT-selective genes in primary brown adipocytes (differentiated for 6 days) upon miR-22 overexpression by lentiviral transduction. n = 4 technical replicates. *P < 0.05, **P < 0.01, and ***P < 0.001 (two-tailed Student's *t*-test). Data are represented as the means ± SDs.

To understand how miR-22 regulates thermogenic function in BAT, we first investigated whether miR-22 is required for brown adipocyte differentiation. To this end, we isolated SVF cells from BAT and subjected them to a standard brown adipogenic differentiation protocol *in vitro*. SVF cells from miR-22 KO and WT mice showed equivalent abilities to develop into mature lipid droplet-containing adipocytes (Figure 4A). In agreement with these findings, inhibitor-induced knockdown of miR-22 did not influence the efficiency of mature lipid droplet-containing adipocyte development (Figure 4B). The levels of pan-adipocyte genes including *Pparγ*, *Fabp4* and *Adipoq* were not altered upon miR-22 inhibition (Figure 4C). In addition, brown preadipocytes infected with empty or miR-22 lentiviral vectors also showed equivalent abilities to develop into mature lipid droplet-containing adipocytes. This was evidenced by comparable of both lipid accumulation and adipogenic genes upon miR-22 overexpression (Figure 4D-F). Together, these data suggest that miR-22 may not participate in the differentiation process of BAT.

However, the brown-selective gene program was significantly downregulated in differentiated SVF cells with miR-22 inhibition at both the mRNA and protein levels (Figure 4G-H). Consequently, miR-22 KO differentiated SVF cells exhibited declines in the OCR, as evidenced by reductions in basal respiration, maximal respiration, space respiration capacity and proton leakage (Figure 4I-J). Moreover, brown-selective genes and OCR increased upon miR-22 overexpression (Figure 4K-M). Taken together, these data demonstrate that miR-22 is required to activate the brown-specific thermogenic program in isolated BAT precursors in a cell-autonomous manner.

Interestingly, we also found that SVF cells from iWAT of miR-22 KO mice exhibited dramatically reduced lipid accumulation (Figure S4A). As indicated by the qRT-PCR results, SVF cells from iWAT of miR-22 KO mice exhibited marked downregulation of adipogenesis markers (Figure S4B). These data suggest that miR-22 may participate in the differentiation process of white adipogenesis *in vitro*.

### Adipose-specific miR-22 AKO mice exhibit impaired browning capacity of WAT

Next, we investigated whether miR-22 plays a role in the development of WAT* in vivo*. The ratios of iWAT and epididymal WAT (eWAT) to body weight, and the size of lipid droplets are comparable between control and miR-22 AKO mice under normal environmental conditions (Figure S5A-D), suggesting that miR-22 does not regulate differentiation of WAT. Subsequently, we used the well-established model of β3AR activation with the highly selective agonist CL316,243 (CL); in this model, chronic CL treatment led to robust browning of WAT. We found that CL treatment resulted in a substantial increase in multilocular adipocytes within iWAT and eWAT, and this effect was accompanied by a robust increase in UCP1^+^ cells (Figure S5C-D). However, the numbers of multilocular adipocytes and UCP1^+^ cells were markedly decreased in iWAT and eWAT from miR-22-AKO mice, compared to controls (Figure S5C-D). Furthermore, a marked decrease in Ucp1 expression was also observed in iWAT and eWAT from AKO mice (Figure S5E-F). Consistently, BAT-selective genes including *Ucp1*, *Prdm16*, *Cidea*, and *Cox5a* were significantly downregulated in iWAT from miR-22-AKO mice at RNA level (Figure S5G). The data suggest that miR-22 is required for generation of beige adipocytes.

To investigate whether miR-22 regulates beige fat development *in vitro*, we isolated SVF cells from iWAT, and subjected them to a standard beige adipogenic differentiation protocol *in vitro*. Oil Red O staining showed that miR-22 loss impaired adipocyte differentiation, leading to decreased lipid droplet accumulation (Figure S5H). In addition, the brown-selective gene program (*Ucp1, Prdm16, Pgclα* and* Elovl3*) was significantly downregulated in differentiated miR-22 KO beige adipocytes, as were the adipocyte marker genes *Fabp4* and *Adipoq* (Figure S5I). The protein level of Ucp1 was also downregulated (Figure S5J). Collectively, the above observations suggest that miR-22 promotes the generation of beige adipocytes in WAT upon cold exposure.

### miR-22 KO mice are resistant to high-fat diet (HFD)-induced insulin resistance

It is well known that dysfunctional BAT can contribute to diet-induced obesity and insulin resistance 34, 35. We thus investigated whether miR-22 deletion results in obesity and insulin resistance under HFD conditions. The body weights of control and miR-22 KO mice were identical before HFD feeding and started to be significantly segregated two weeks after HFD feeding (Figure S6A). Unexpectedly, global miR-22 KO mice exhibited resistance to HFD-induced obesity. With regard to serum metabolites, the plasma glucose and total cholesterol levels were significantly lower in the miR-22 KO mice than in the WT mice (Figure S6B-C). Moreover, miR-22 KO mice displayed better glucose tolerance and increased insulin sensitivity (Figure S6D-E). We further found that liver weight and hepatic fat accumulation in miR-22 KO mice upon HFD were decreased compared with those in WT mice (Figure S6F-H). It suggests that insulin sensitivity is improved in miR-22 KO mice upon HFD feeding compared with those in WT mice.

To test whether the phenotypes observed upon miR-22 loss originated in BAT, we used adipose-specific AKO mice for further verification. The weight gain of adipose-specific miR-22-AKO mice was similar to that of control mice fed a HFD (Figure S6I). Despite similar body masses, adipose-specific miR-22 AKO mice were less intolerant to glucose and more sensitive to an intraperitoneal insulin tolerance test than control mice (Figure S6J-K). Similar to constitutive KO mice, liver weight and hepatic fat accumulation in miR-22 AKO mice upon HFD were decreased compared with those in control mice (Figure S6L-M). Overall, these results indicate that miR-22 deficiency improves glucose tolerance and insulin sensitivity and protects against liver steatosis under HFD conditions.

### miR-22 regulates glycolysis in BAT

**Figure 5:**
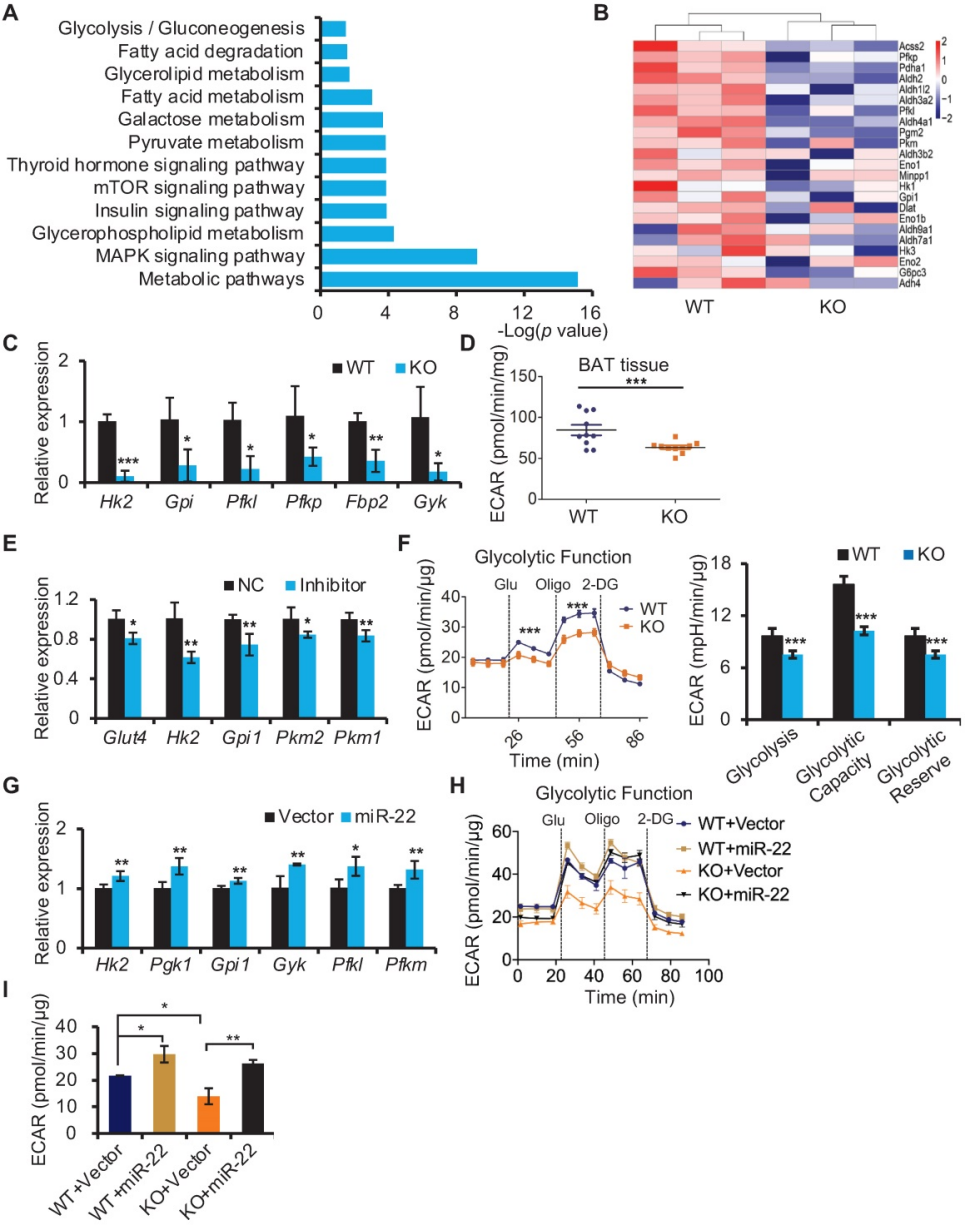
miR-22 promotes glycolysis in BAT. (A) KEGG pathway analysis of the downregulated genes between miR-22 KO mice and their littermate controls. n =3 biological replicates. (B) Heatmaps showing differentially expressed genes involved in glycolysis. (C) qRT-PCR analysis for the indicated glycolytic genes in BAT from WT and miR-22 KO mice. n = 3 biological replicates. (D) ECAR of BAT isolated from WT and miR-22 KO mice (n = 10). (E) qRT-PCR analysis for the indicated glycolytic genes in differentiated primary brown adipocytes in response to miR-22 inhibitor (day 6). n = 3 technical replicates. (F) ECAR in WT and miR-22 KO differentiated primary brown adipocytes (day 6), Glucose, Oligomycin (oligo) and 2-DG were added at the time points indicated by the dashed lines. (G) qRT-PCR analysis for the indicated glycolytic genes in primary brown adipocytes (differentiated for 6 days) upon miR-22 overexpressed by lentiviral transduction. n = 4 technical replicates. (H-I) ECAR in WT and miR-22 KO differentiated primary brown adipocytes (day 6) after miR-22 overexpression. *P < 0.05, **P < 0.01, and ***P < 0.001 (two-tailed Student's *t*-test). Data are represented as the means ± SEMs (F and G) and others as the means ± SDs.

To gain insight into the molecular mechanism underlying the compromised thermogenic function of BAT in miR-22 KO mice, we performed transcriptome profile analysis on BAT from WT (n = 3) and miR-22 KO (n = 3) mice and found 1791 downregulated genes and 1065 upregulated genes in miR-22 KO mice (Figure S7A). In agreement with the impaired thermogenic function, most thermogenesis-related genes were downregulated in miR-22 KO mice (Figure S7B). With respect to the downregulated genes, KEGG pathway analysis showed that the downregulated genes were significantly enriched in pyruvate metabolism, fatty acid metabolism, and fatty acid degradation (Figure 5A). Of interest, glycolysis was also among the enriched KEGG pathway terms (Figure 5A). It is worth noting that glycolysis can compensate for the loss of mitochondrial ATP production due to heat-generating mitochondrial uncoupling in response to cold exposure 36. An array of genes involved in the glycolytic pathway was downregulated in miR-22 KO mice (Figure 5B). The levels of *Hk2*, *Gpi*, *Pfkl*, *Pfkp*,* Fbp2* and *Gyk* were selectively confirmed by qRT-PCR analyses (Figure 5C). Furthermore, we employed a Seahorse XF analyzer (Agilent Technologies) to measure the ECAR as an index of glycolytic activity. The ECAR of BAT isolated from miR-22 KO mice was significantly lower than that of BAT isolated from WT mice (Figure 5D). Collectively, these data indicate that the glycolytic activity is suppressed in BAT in response to miR-22 deficiency. Furthermore, an inhibitory effect on the glycolytic activity was also found in adipocyte-specific miR-22 AKO and miR-22 BKO mice (Figure S7C-E), suggesting that miR-22 has a BAT specific function.

In line with the *in vivo* findings, differentiated SVF from brown preadipocytes with inhibitor-mediated knockdown of miR-22 also exhibited reduced expression levels of genes involved in glycolysis (Figure 5E). Decreases in ECAR were also found in differentiated miR-22 KO SVF cells, as evidenced by reductions in the basal glycolytic capacity and maximum glycolytic capacity (Figure 5F). Conversely, the glycolytic genes and ECAR were increased upon miR-22 overexpression (Figure 5G-5I). Taken together, these data demonstrate that miR-22 promotes glycolytic activity in BAT.

### miR-22 promotes thermogenesis and glycolysis by directly suppressing *Hif1an*

**Figure 6:**
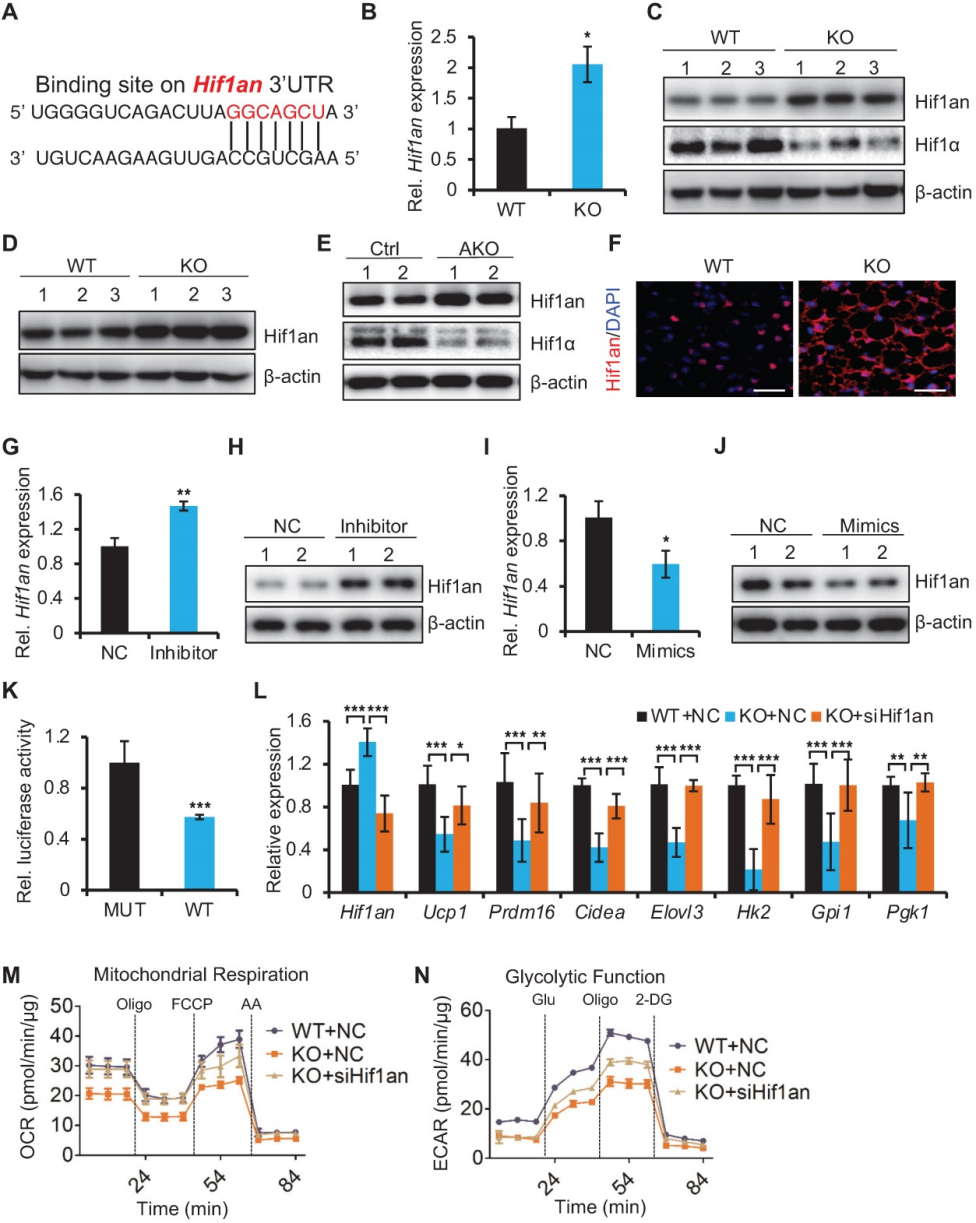
miR-22 promotes thermogenesis and glycolysis by directly suppressing Hif1an. (A) The miR-22 binding site on the *Hif1an* 3'UTR. (B) qRT-PCR analysis for *Hif1an* in BAT from WT and miR-22 KO mice. n = 3 biological replicates. (C) Western blots of Hif1an and Hif1α in BAT from WT and miR-22 KO mice. β-actin was used as a loading control. (D) Western blots of Hif1an in differentiated primary brown adipocytes (day 6). β-actin was used as a loading control. (E) Western blots of Hif1an and Hif1α in BAT from control and miR-22 AKO mice. β-actin was used as a loading control. (F) Immunofluorescence for Hif1an in BAT from WT and miR-22 KO mice Scale bar: 50 µm. (G and I) qRT-PCR analysis for *Hif1an* in differentiated primary brown adipocytes upon miR-22 inhibitor or mimics treatments. n = 3 technical replicates. (H and J) Western blots for Hif1an in differentiated primary brown adipocytes upon miR-22 inhibitor or mimics treatments. β-actin was used as a loading control. (K) Ratios of the luciferase activity of miR-22 mimics versus that of scramble RNA in WT and mutant 3'UTR constructs based on 3 independent experiments. (L) qRT-PCR analysis for the indicated genes in thermogenic and glycolytic pathways in differentiated primary brown adipocytes (day 6) upon Hif1an siRNA treatments. n = 3 technical replicates. Data were analyzed by two-way ANOVA followed by Tukey's test. (M-N) OCR (M) and ECAR (N) in differentiated primary brown adipocytes (day 6) at indicated conditions. *P < 0.05, **P < 0.01, and ***P < 0.001 (The data are analyzed by two-tailed Student's *t*-test unless specifically noted). Data are represented as the means ± SEMs (M and N) and others as the means ± SDs.

To understand how miR-22 regulates thermogenesis and glycolysis, we analyzed miR-22 binding sites in the 3'UTRs of transcripts encoding inhibitors of the glycolytic pathway using TargetScan algorithms. We found that the 3'UTR of the *Hif1an* transcript includes miR-22 binding sites (Figure 6A). As an asparagine hydroxylase, Hif1an can hydroxylate hypoxia-inducible factor 1a (Hif1α) to suppress its transcriptional activity, while Hif1α is a critical regulator of glycolysis 37-39. The expression levels of thermogenic and glycolytic genes in brown fat decreased upon Hif1α knockdown (Figure S8A). Moreover, the Hif1an-knockout mice exhibited increased Ucp1 expression and elevated EE 40. To determine whether *Hif1an* is a direct miR-22 target, we quantified the expression levels of *Hif1an* in BAT from miR-22 KO mice. *Hif1an* was markedly upregulated in BAT from miR-22 KO mice at both the mRNA (Figure 6B) and protein levels (Figure 6C), as well as in primary SVF-derived differentiated precursors from miR-22 KO mice (day 6) (Figure 6D). Upregulation of Hif1an was also observed in BAT from miR-22 AKO mice (Figure 6E), and upregulation of Hif1an was further confirmed by immunofluorescence (Figure 6F). We also observed that the protein levels of Hif1α were decreased both in BAT from both global and adipocyte-specific miR-22-knockout mice (Figure 6C and 6E). An *in vitro* assay showed that *Hif1an* was markedly upregulated in response to miR-22 inhibitor treatment (Figure 6G-H), while the glycolytic activity was suppressed (Figure 5E). Conversely, *Hif1an* was downregulated upon miR-22 mimics treatment (Figure 6I-J). Furthermore, 3'UTR-luciferase reporter assays revealed that miR-22 mimics significantly repressed WT *Hif1an* 3'UTR activity, while mutation of the miR-22 3'UTR binding site in these constructs abrogated this repression (Figure 6K).

Next, we asked whether *Hif1an* functionally mediates the observed biological function of miR-22. Using a mature brown adipocyte transfection technique 28, we knocked down *Hif1an* in differentiated miR-22 KO SVF cells with siRNA. The inhibition of *Hif1an* significantly rescued the expression levels of the brown fat thermogenic and glycolytic genes (Figure 6L), and the decrease in OCR and ECAR induced by miR-22 deficiency was also rescued by Hif1an knockdown (Figure 6M-6N and Figure S8B-C), suggesting *Hif1an* as a functional target of miR-22. Together, these findings demonstrate that miR-22 promotes thermogenesis and glycolytic activity in BAT by directly suppressing *Hif1an*.

### miR-22 activates mTORC1 signaling in BAT

**Figure 7:**
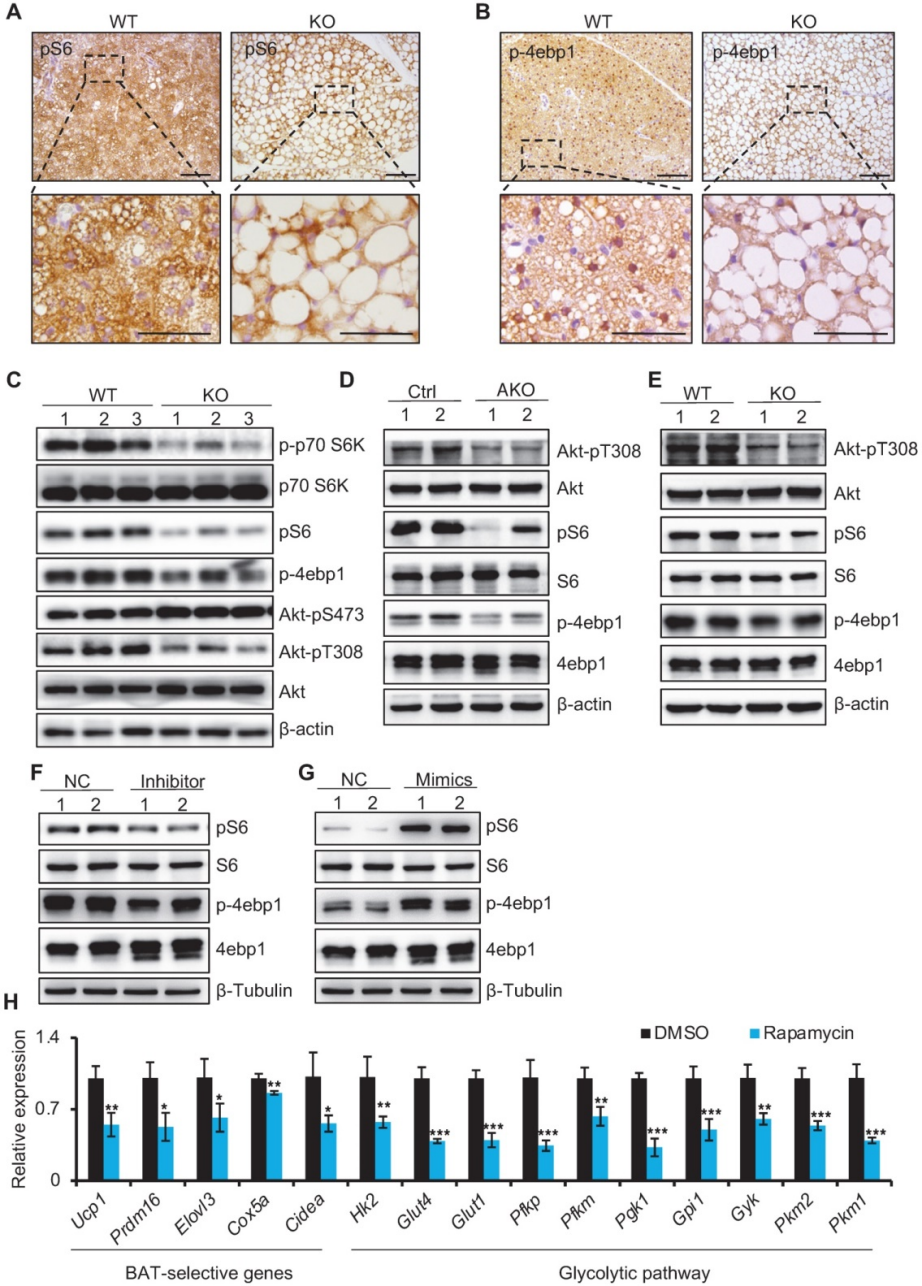
miR-22 activates mTORC1 signaling in BAT. (A-B) Immunohistochemistry for pS6 (A) and p-4ebp1 (B) in BAT from WT and miR-22 KO mice. The dashed boxes indicate the high magnification images in the bottom panels. Scale bar: 100 µm. (C) Western blots of selective proteins in mTORC1 signaling pathway in BAT from WT and miR-22 KO mice. β-actin was used as a loading control. (D) Western blots of selective proteins in mTORC1 signaling pathway in BAT from control and miR-22 AKO mice. β-actin was used as a loading control. (E) Western blots of selective proteins in mTORC1 signaling pathway in primary brown adipocytes (day 6) from WT and miR-22 KO mice. β-Tubulin was used as a loading control. (F-G) Western blots for pS6 and p-4ebp1 in differentiated primary brown adipocytes upon miR-22 inhibitor (F) or mimics (G) treatments. β-Tubulin was used as a loading control. (H) qRT-PCR analysis for the indicated genes in thermogenic and glycolytic pathways in differentiated primary brown adipocytes (day 6) upon rapamycin treatment (10 nM, 12 H). n = 3 technical replicates. *P < 0.05, **P < 0.01, and ***P < 0.001 (two-tailed Student's *t*-test). Data are represented as the mean ± SD.

Previous studies have indicated that mTORC1 is activated in brown adipocytes, functioning in both BAT thermogenesis and glycolytic activity 13. Strikingly, the mTOR signaling pathway was among the enriched KEGG pathway terms (Figure 5A). We thus examined the status of mTORC1 signaling. mTORC1 activity was reduced in BAT from miR-22 KO mice, as evidenced by reductions in the pS6 and p-4ebp1 levels (indicators of mTORC1 activity) (Figure 7A-B). Consistently, the phosphorylation levels of S6, 4ebp1, S6k, and Akt-T308 in BAT from miR-22 KO mice were significantly downregulated compared with that from WT mice, while the phosphorylation levels of Akt-S473 were upregulated (Figure 7C). Compromised mTORC1 activity was also found in BAT from miR-22 AKO mice and in differentiated primary brown adipocytes from miR-22-KO mice (day 6) (Figure 7D-E). To test whether miR-22 can directly activate the mTORC1 pathway, we examined the effects of miR-22 alterations on the mTORC1 signaling status in differentiated primary brown adipocytes. Western blot assays showed that miR-22 inhibitor treatment decreased the protein levels of pS6 and p-4ebp1, whereas miR-22 mimics treatment increased them (Figure 7F-G). In line with this, thermogenic genes and glycolytic activity were impaired in differentiated primary adipocytes after rapamycin treatment (Figure 7H). Collectively, these results demonstrate that miR-22 activates the mTORC1 signaling pathway in BAT.

### miR-22 directly targets negative regulators of mTORC1 signaling

**Figure 8:**
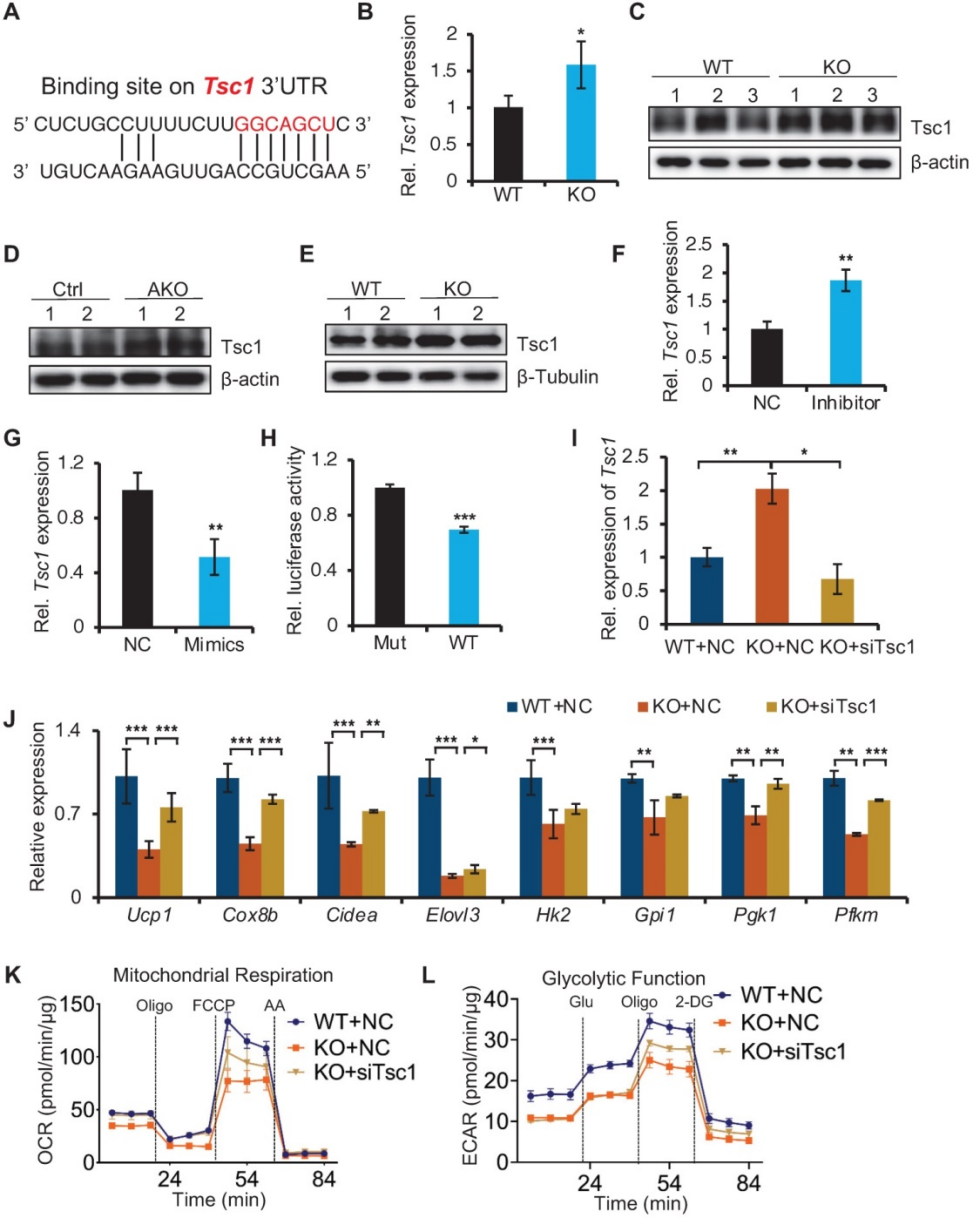
Identification of miR-22 direct targets in regulating mTORC1 signaling. (A) The miR-22 binding sites on the *Tsc1* 3'UTRs. (B) qRT-PCR analysis for *Tsc1* in BAT from WT and miR-22 KO mice. n = 3 biological replicates. (C) Western blots of Tsc1 in BAT from WT and miR-22 KO mice. β-actin was used as a loading control. (D) Western blots of Tsc1 in BAT from control and miR-22 AKO mice. β-actin was used as a loading control. (E) Western blots of Tsc1 in differentiated primary brown adipocytes (day 6) from control and miR-22 KO mice. β-Tubulin was used as a loading control. (F-G) qRT-PCR analysis for *Tsc1* in differentiated primary brown adipocytes after miR-22 inhibitor (F) or mimics (G) treatment. n = 3 technical replicates. (H) Ratios of the luciferase activity of miR-22 mimics versus that of scramble RNA in WT and mutant 3'UTR constructs based on 3 independent experiments. (I) qRT-PCR analysis for *Tsc1* in differentiated primary brown adipocytes (day 6) upon *Tsc1* siRNA treatments. n = 3 technical replicates. (J) qRT-PCR analysis for the indicated genes in thermogenic and glycolytic pathways in differentiated primary brown adipocytes (day 6) upon *Tsc1* siRNA treatments. n = 3 technical replicates. Data were analyzed by two-way ANOVA followed by Tukey's test. (K-L) OCR (K) and ECAR (L) in differentiated primary brown adipocytes (day 6) at indicated conditions. *P < 0.05, **P < 0.01, and ***P < 0.001 (The data are analyzed by two-tailed Student's *t*-test unless specifically noted). Data are represented as the means ± SEMs (K and L) and others as the means ± SDs.

To understand how miR-22 regulates the mTORC1 pathways, we analyzed the miR-22 binding sites in the 3'UTRs of transcripts encoding the regulators of these pathways. The target prediction tool of TargetScan revealed that the mTORC1 pathway antagonists Tsc1 feature miR-22 binding sites (Figure 8A), suggesting that it is potential target of miR-22. *Tsc1* in BAT from miR-22 KO mice were significantly upregulated compared with that in BAT from WT mice at both the RNA and protein levels (Figure 8B-C). Upregulation of Tsc1 was also observed in BAT from miR-22 AKO mice, and in differentiated primary brown adipocytes from miR-22 KO mice (Figure 8D-E). The *Tsc1* level in differentiated primary adipocytes increased in response to miR-22 inhibitor treatment (Figure 8F). Conversely, the level of *Tsc1* decreased upon miR-22 mimics treatment (Figure 8G). Next, we validated the direct repression of target transcripts by miR-22 activity using WT-3'UTR-luciferase constructs for *Tsc1*. Mutation of the miR-22 3'UTR binding site in these constructs abrogated the observed repression (Figure 8H). In line with this finding, the expression levels of Tsc1 declined over the course of brown adipocyte differentiation, exhibiting a negative correlation with the miR-22 levels (Figure S9A). To further verify whether mTORC1 signaling mediated miR-22 function, we activated mTORC1 signaling in miR-22-KO primary brown adipocytes by inhibiting Tsc1. We found that the decrease in thermogenic and glycolytic genes caused by miR-22 deletion was rescued by inhibition of Tsc1 (Figure 8I-J and Figure S9B). Furthermore, the reductions in the OCR and ECAR induced by miR-22 deletion were also rescued by Tsc1 knockdown (Figure 8K-L and Figure S9C-D). Taken together, these findings demonstrate that miR-22 activates mTORC1 signaling by directly suppressing *Tsc1*, thereby modulating the glycolytic activity and thermogenic function of BAT.

## Discussion

In this study, we demonstrated that miR-22 promotes BAT thermogenic function by directly modulating glycolysis and thermogenic genes (Figure S10). Previous studies have identified several miRNAs, including miR-133, miR-155, miR-455 and miR-378, as regulators of BAT thermogenesis 21, 41-43. This work adds miR-22 as a novel regulator, further suggesting the importance of miRNAs in BAT thermogenesis. Interestingly, almost all previously reported miRNAs regulate BAT thermogenic function by modulating BAT differentiation, while miR-22 directly modulates glycolysis and thermogenic genes. It appears that miRNAs can regulate BAT thermogenic function in distinct ways.

In this study, no significant changes in serum biochemical indices or metabolic cage parameters were found between Ctrl and AKO mice, although significant alterations were observed in constitutive KO mice. This could be explained by the fact that metabolic changes found in constitutive KO mice could be caused by miR-22 deletion in tissues other than adipose tissues, and that no significant alteration in AKO mice is compensated by other metabolism-relevant tissues. Indeed, it has been reported that miR-22 promotes lipid metabolism in skeletal muscles 44, and that elevated hepatic miR-22-3p expression impairs gluconeogenesis by silencing the Wnt-responsive transcription factor tcf7 45. It appears that miR-22 is extensively important in metabolism-relevant organs. Future studies on the role of miR-22 in other organs will be merited.

The mTORC1 pathway is a well-known engine of anabolic metabolism in different types of cells 46. Here, we demonstrated that miR-22 regulates BAT thermogenesis by directly activating the mTORC1 signaling pathway. In line with our findings, previous studies have shown that mice with mTORC1 impairment mediated by either adipocyte-specific deletion of Raptor or pharmacological inhibition (rapamycin treatment) are cold intolerant, as such impairment blocks the expression of thermogenic genes and the expansion of beige adipocytes in WAT 14, 15. It has been demonstrated that mTORC1 is dispensable for brown adipocyte terminal differentiation 47, 48. Consistently, miR-22 does not affect the differentiation of brown fat progenitor cells. However, miR-22 is critical for activating the brown-specific thermogenic program in BAT. Thus, we have identified miR-22 as an important regulator of the mTORC1 signaling pathway in adipocytes that mediates cold exposure-induced heat energy production.

Another striking finding was that miR-22 enhanced BAT thermogenesis by modulating glycolysis via direct regulation of Hif1an. Consistent with our findings, inhibition of glycolysis in BAT has been found to result in thermogenesis failure in mice and to increase mouse sensitivity to cold exposure 9, 49. The miR-22 target gene Hif1an is an inhibitor of Hif1α, which is a critical regulator of glycolysis 50. Knockdown of Hif1α diminishes glycolytic capacity, glucose uptake, lactate secretion and β-adrenaline-stimulated oxygen consumption in brown adipocytes 39, while the loss of Hif1an leads to increased Ucp1 expression and elevated EE 40, supporting the notion that miR-22 enhances BAT thermogenesis by regulating glycolysis. Interestingly, miR-22-induced mTORC1 activity also increases glycolytic flux by indirectly activating the transcription and translation of HIF1α 11. In agreement with this result, the glycolytic activity is impaired in differentiated primary adipocytes in response to rapamycin treatment. In summary, we propose a model in which miR-22 functions as an important cell-intrinsic modulator of glycolysis in BAT via two distinct pathways, the mTORC1 and Hif1an pathways.

Given that improvement of BAT thermogenic capacity might benefit systemic metabolic homeostasis, we hypothesized that miR-22 deletion in adipocytes sensitizes mice to metabolic damage promoted by a HFD. However, the deletion of miR-22 in adipocytes does not predispose HFD-fed mice to systemic metabolic dysregulation. In contrast, glucose tolerance and insulin sensitivity were increased in both global and adipocyte-specific miR-22 KO mouse models. Our results are agreement with recent evidence from studies on several adipose-specific KO mouse models, such as lysine-specific demethylase 1 (Lsd1) 51, adipose triglyceride lipase (ATGL) 52, and mitofusin2 (Mfn2) 53, 54 adipose-specific KO mouse models, all of which revealed impaired thermogenesis in BAT but resistance to HFD-induced insulin resistance. Recent studies have shown that miR-22-3p antagonism improves insulin sensitivity and that loss of miR-22 prevents HFD-induced metabolic disorders 45, 55. One explanation could be that miR-22-deficient adipocytes might secrete some signaling molecules that potentially activate energy expenditure in other metabolic organs, such as the liver and muscle 56, 57. Indeed, fibroblast growth factor 21 (FGF21) is a master metabolic regulator that serves as an important paracrine signaling molecule with remarkable ability to reverse diabetes and obesity 58. The levels of miR-22 are inversely correlated with those of FGF21 and FGFR1 in human and mouse fatty livers, and miR-22 inhibition reduces hepatic steatosis via FGF21 and FGFR1 induction 59. In addition, brown adipose tissue is a source of systemic FGF21 60. Thus, the indirect negative regulation of FGF21 by miR-22 could cause insulin resistance. In agreement with this idea, we found that hepatic fat accumulation is decreased in both global and adipocyte-specific miR-22-KO mice upon HFD feeding. Thus, the miR-22 KO mice phenotype against HFD-induced insulin resistance might result from systemic metabolism compensation in other organs.

## Conclusion

In summary, using global and conditional miR-22 KO mouse models, we found that miR-22 deficiency led to suppressed thermogenic gene expression in BAT, BAT whitening, decreased EE and impaired cold adaptation. Mechanistically, miR-22 directly targets *Tsc1* to activate the mTORC1 signaling pathway and concomitantly directly suppresses *Hif1an*, an inhibitor of Hif1α, to promote glycolysis and maintain thermogenesis.

## Supplementary Material

Supplementary figures and tables.
