# Supplementary material for: MiR-22 modulates brown adipocyte thermogenesis by synergistically activating the glycolytic and mTORC1 signaling pathways
Source: Theranostics. 2021 Jan 25;11(8):3607–23. doi: 10.7150/thno.50900 (PMC7914365; doi:10.7150/thno.50900)
Supplement: Supplementary file 1 — Supplementary figures and tables. [file thnov11p3607s1.pdf]

Supplemental materials

MiR-22 modulates brown adipocyte thermogenesis by synergistically activating the glycolytic and mTORC1 signaling pathways

Pengbo Lou[1#], Xueyun Bi[1#], Yuhua Tian[1#], Guilin Li[1], Qianqian Kang[1], Cong Lv[1], Yongli Song[1], Jiuzhi Xu[1], Xiaole Sheng[1], Xu Yang[1], Ruiqi Liu[1], Qingyong Meng[1], Fazheng Ren[2], Maksim V. Plikus[3], Bin Liang[4], Bing Zhang[5*], Huiyuan Guo[2*], Zhengquan Yu[1*]

1. State Key Laboratories for Agrobiotechnology and Key Laboratory of Precision Nutrition and Food Quality, Ministry of Education, Department of Nutrition and Health, College of Biological Sciences, China Agricultural University, Beijing, China, 100193;
2. Key Laboratory of Precision Nutrition and Food Quality, Ministry of Education, Department of Nutrition and Health, College of Food Sciences and nutritional engineering, China Agricultural University, Beijing, China, 100083;
3. Department of Developmental and Cell Biology, Sue and Bill Gross Stem Cell Research, Center for Complex Biological Systems, University of California, Irvine, Irvine, CA 92697, USA;
4. Center for Life Sciences, School of Life Sciences, Yunnan University, Kunming, Yunnan, China, 650091;
5. College of Veterinary Medicine, China Agricultural University, Beijing, China

[#]Authors share the co-first authorship.

*Co-corresponding authors: zyu@cau.edu.cn (Zhengquan Yu, lead contact); guohuiyuan@cau.edu.cn (Huiyuan Guo); zhangdb@cau.edu.cn (Bing Zhang).

Supplemental materials include 10 figures and 3 tables.

Figure S1. miR-22 is highly expressed in brown fat and upregulated after cold treatment.

Figure S2. No changes in serum biochemical indices or metabolic cage indices were observed in miR-22 AKO mice on a chow diet at RT.

Figure S3. Whitening of BAT in miR-22 AKO and BKO mice.

Figure S4. miR-22 is required for WAT adipogenesis *in vitro*.

Figure S5. Adipose-specific miR-22 AKO mice exhibit defective browning capacity of WAT.

Figure S6. miR-22 KO mice are protected against HFD-induced insulin resistance.

Figure S7. Glycolysis is suppressed in BAT from miR-22 conditional knockout mice.

Figure S8. miR-22 promotes thermogenesis and glycolysis by directly suppressing Hif1an.

Figure S9. Identification of miR-22 direct targets in regulating mTORC1 signaling.

Figure S10. The working model of miR-22 in regulating BAT thermogenesis.

Table S1. Primers used in the present study.

Table S2. siRNA or inhibitor used in the present study.

Table S3. The antibodies used in this study.

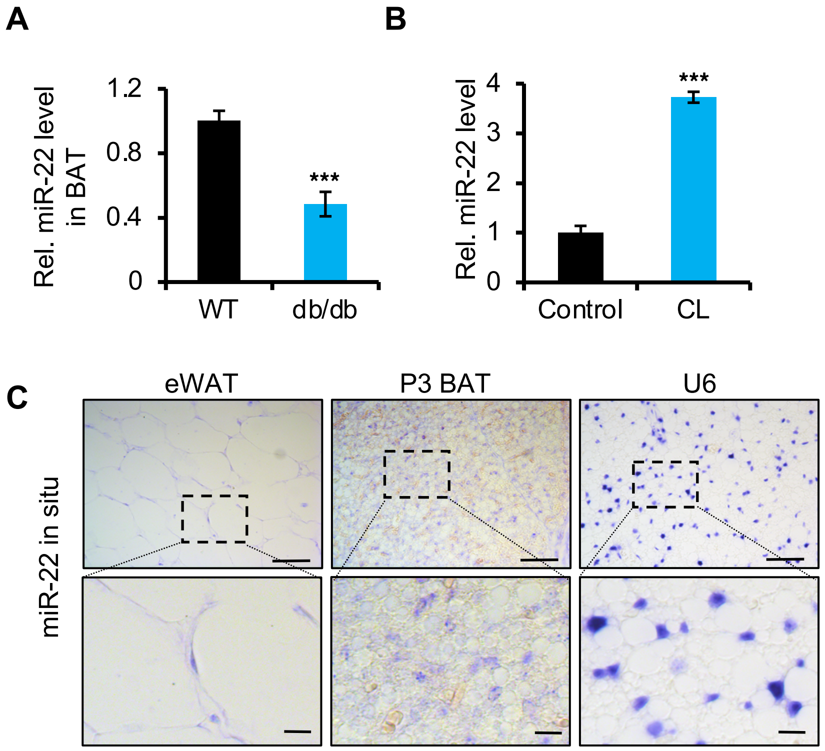

Figure S1. miR-22 is highly expressed in brown fat and upregulated after cold treatment. (A) qRT-PCR analysis for miR-22 in BAT of wild type and db/db mice. n = 3 biological replicates. (B) qRT-PCR analysis for miR-22 in primary brown adipocytes (differentiated for 6 days) in response to CL (CL316,243) treatment (0.5 µM, 6 H). n = 3 technical replicates. (C) In situ hybridization for miR-22 at indicated conditions. Top panel, representative low magnification image (Scale bar: 50 µm); bottom panels, high magnification images indicated by dashed boxes in top panel image (Scale bar: 10 µm), U6 used as a positive control, eWAT: epididymal WAT. *$P < 0.05$, **$P < 0.01$, and ***$P < 0.001$ (two-tailed Student's *t*-test).

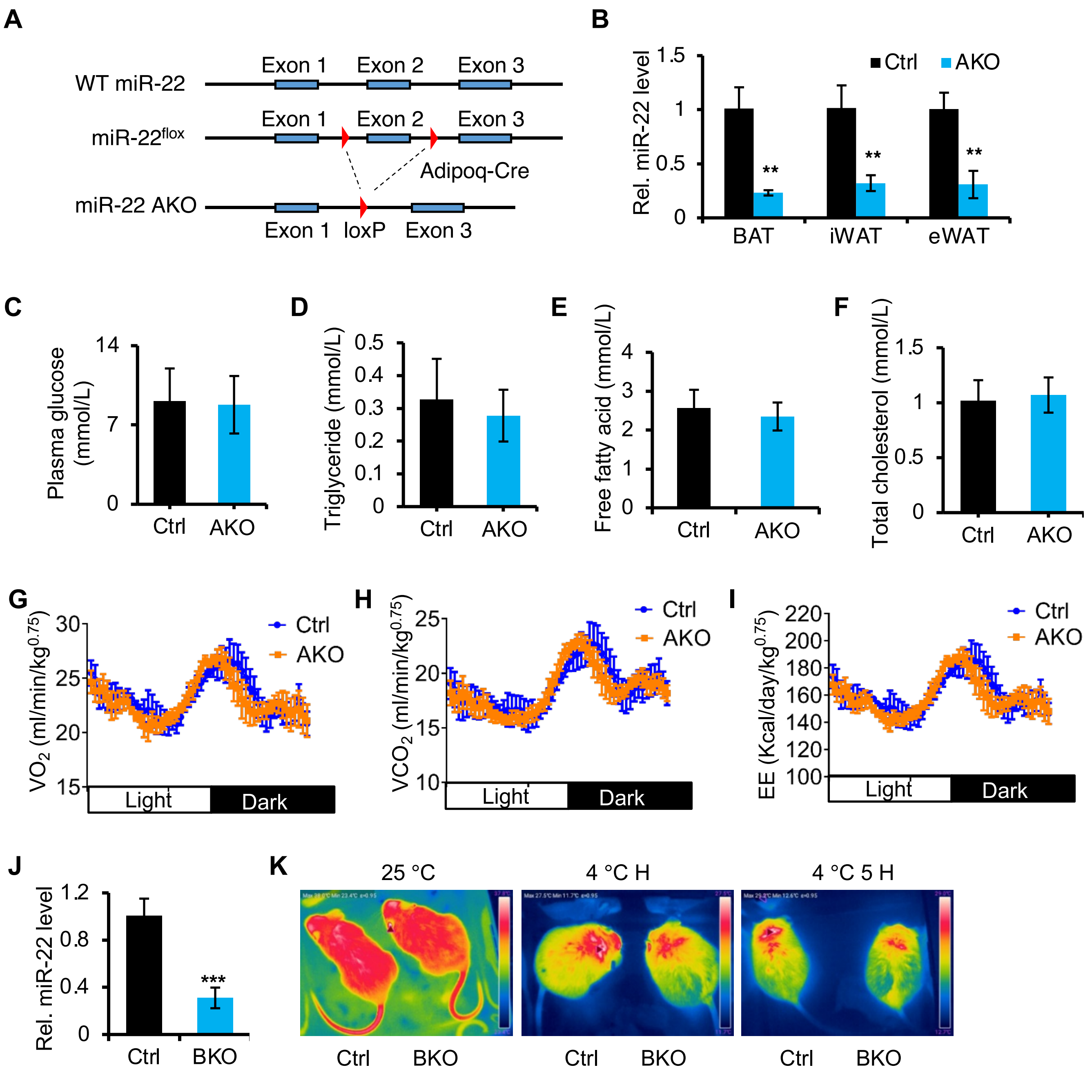
A
WT miR-22
miR-22flox
miR-22 AKO
Exon 1
Exon 2
Exon 3
Adipoq-Cre
loxP
B
Rel. miR-22 level
Ctrl
AKO
1.5
1
0.5
0
**
BAT
iWAT
eWAT
C
Plasma glucose (mmol/L)
14
7
0
Ctrl
AKO
D
Triglyceride (mmol/L)
0.5
0.4
0.3
0.2
0.1
0
E
Free fatty acid (mmol/L)
4
3
2
1
F
Total cholesterol (mmol/L)
G
VO2 (ml/min/kg0.75)
30
25
20
15
Light
Dark
H
VCO2 (ml/min/kg0.75)
10
I
EE (Kcal/day/kg0.75)
220
200
180
160
140
120
100
J
Rel. miR-22 level
1.2
0.8
0.4
***
BKO
K
25 °C
4 °C H
4 °C 5 H

Figure S2. No changes in serum biochemical indices or metabolic cage indices were observed in miR-22 AKO mice on a chow diet at RT. (A) Schematics of generating miR-22 AKO mouse. (B) qRT-PCR analysis for miR-22 in BAT, iWAT and eWAT from miR-22 AKO mice and littermate controls (n = 3). (C-F) The levels of glucose (C), triglyceride (TAG) (D), free fatty acid (E) and total cholesterol (F) in the serum from Ctrl (n = 8) and AKO (n = 8) male mice under 16-hour fasted conditions. (G-I) Indirect calorimetry analysis of oxygen consumption (VO_2) (G), exhaled carbon dioxide (VCO_2) (H), and EE (I) in WT (n = 8) and AKO (n = 8) mice. (J) qRT-PCR analysis for miR-22 in BAT from miR-22 BKO mice and littermate controls (n = 3). (K) Representative thermal images of miR-22 BKO mice (n = 3) and their littermate controls (n = 3) at the indicated conditions. *$P < 0.05$, **$P < 0.01$, and ***$P < 0.001$ (two-tailed Student's *t*-test). Data are represented as the means ± SEMs (G-I) and others as the means ± SDs. BKO: brown adipocyte-specific miR-22 knockout mice, *Ucp1-Cre*; *miR-22*$^{f/f}$.

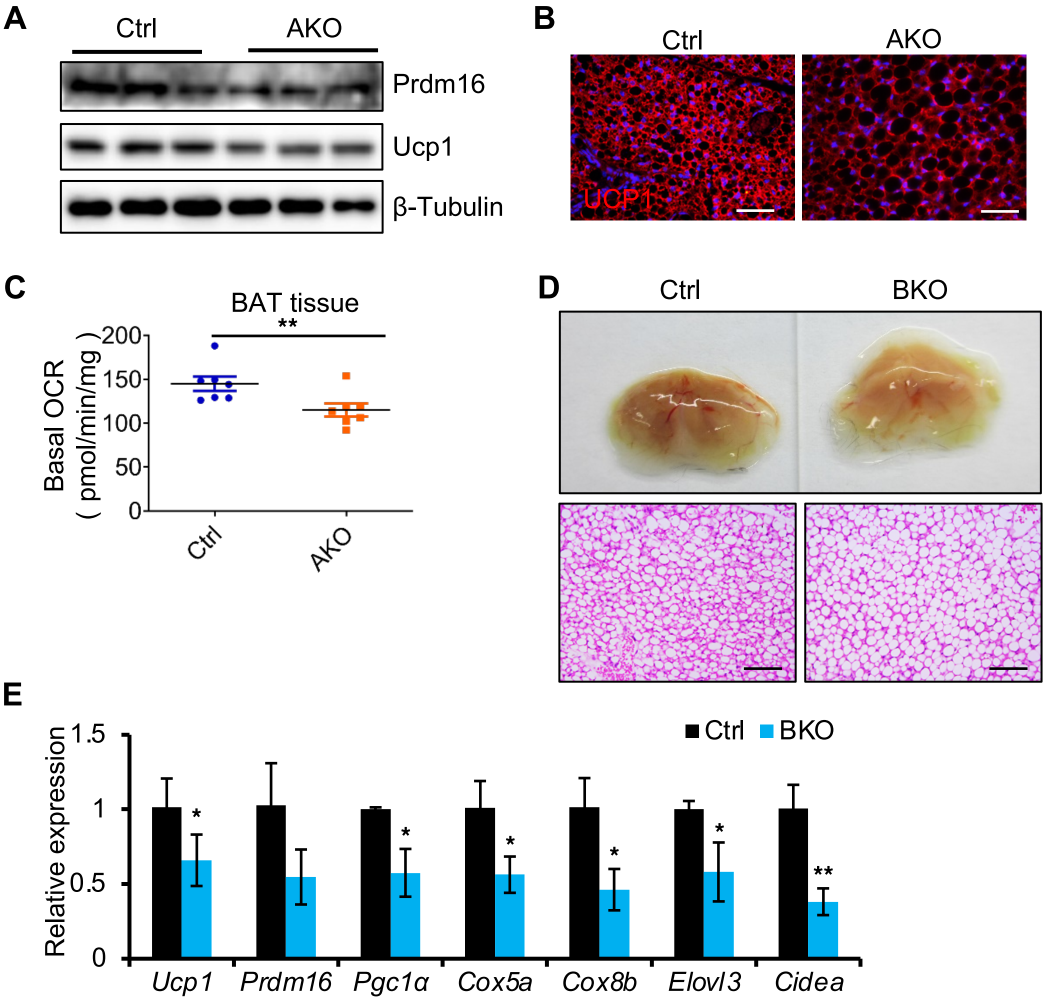

Figure S3. Whitening of BAT in miR-22 AKO and BKO mice. (A) Western blots for Ucp1 and Prdm16 in BAT from control and miR-22 AKO mice. β-Tubulin was used as a loading control. (B) Immunofluorescence of Ucp1 in BAT from control and miR-22 AKO mice. Scale bar: 50 μm. (C) O_2 consumption of isolated BAT from control and miR-22 AKO mice. n = 7 biological replicates. (D) Macroscopic and histological images of control and miR-22 BKO mice BAT at the age of 10 weeks. n = 3 biological replicates. Scale bar: 50 μm. (E) qRT-PCR analysis for BAT-selective genes in BAT from control and miR-22 BKO mice at the age of 10 weeks. n = 3 biological replicates. *$P < 0.05$, **$P < 0.01$, and ***$P < 0.001$ (two-tailed Student's *t*-test).

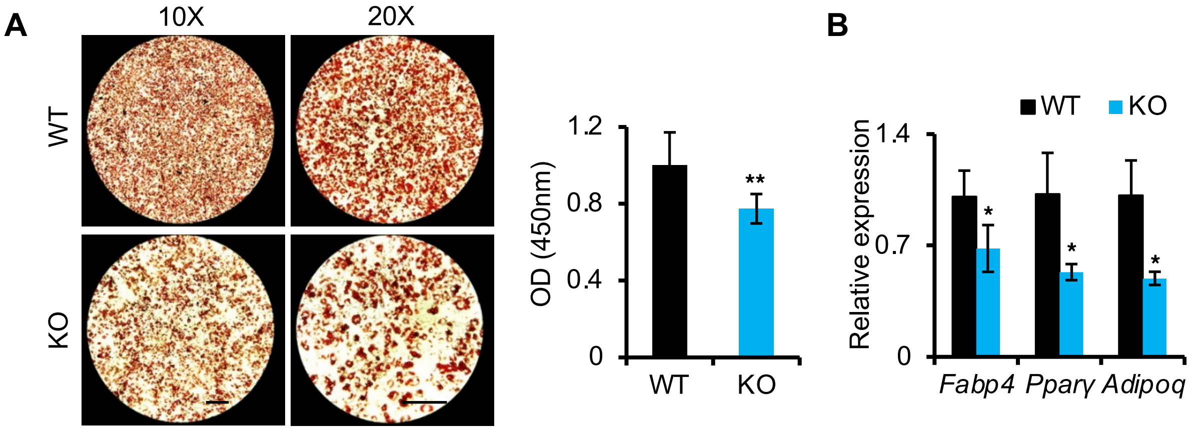

Figure S4. miR-22 is required for WAT adipogenesis *in vitro*. (A) Oil red O staining in differentiated primary white adipocytes (day 6) (Scale bar: 220 μm) and quantification of Oil red O dye by spectrophotometer at 450 nm. n = 3 technical replicates. (B) qRT-PCR analysis for pan-adipocyte genes in differentiated primary white adipocytes (day 6). n = 3 technical replicates. **P* < 0.05, ***P* < 0.01, and ****P* < 0.001 (two-tailed Student's *t*-test).

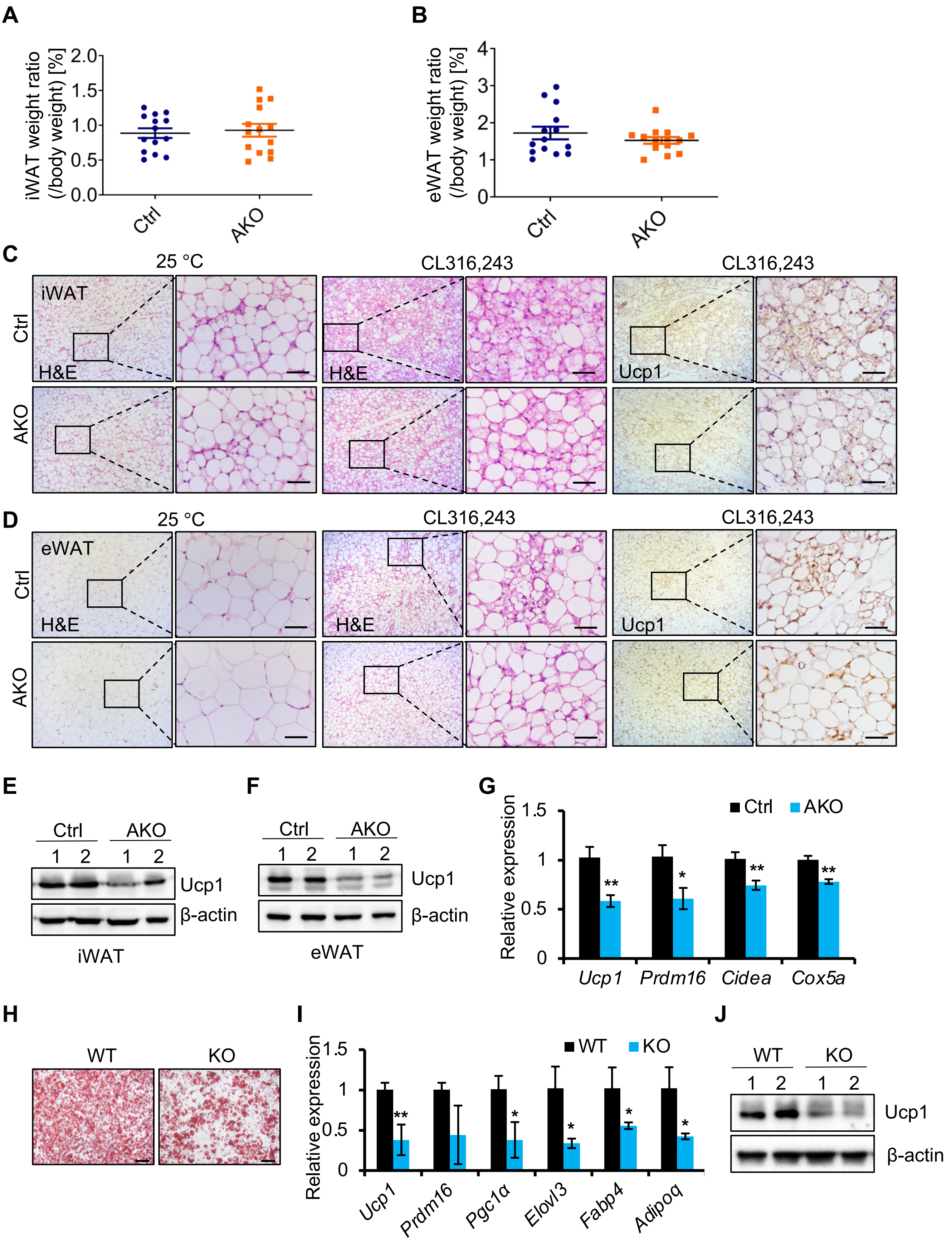
A
iWAT weight ratio (/body weight) [%]
2.0
1.5
1.0
0.5
0.0
Ctrl
AKO
B
eWAT weight ratio (/body weight) [%]
4
3
2
1
0
Ctrl
AKO
C
25 °C
CL316,243
CL316,243
iWAT
Ctrl
AKO
H&E
H&E
Ucp1
D
25 °C
CL316,243
CL316,243
eWAT
Ctrl
AKO
H&E
H&E
Ucp1
E
Ctrl
AKO
1 2 1 2
Ucp1
β-actin
iWAT
F
Ctrl
AKO
1 2 1 2
Ucp1
β-actin
eWAT
G
Relative expression
1.5
1
0.5
0
Ctrl
AKO
**
*
**
**
Ucp1
Prdm16
Cidea
Cox5a
H
WT
KO
I
Relative expression
1.5
1
0.5
0
WT
KO
**
*
*
*
*
Ucp1
Prdm16
Pgc1α
Elovl3
Fabp4
Adipoq
J
WT
KO
1 2 1 2
Ucp1
β-actin

Figure S5. Adipose-specific miR-22 AKO mice exhibit defective browning capacity of WAT. (A-B) Ratios of iWAT (A), and eWAT (B) weight to body weight (n = 14) in control and AKO mice. (C-D) Histochemical staining by H&E (left and middle panels) and Ucp1 immunohistochemical staining (right panel) for iWAT (C) and eWAT (D) section from control and AKO mice. Scale bar: 50 μm. (E-F) Western blots for Ucp1 in iWAT (E) and eWAT (F) from AKO mice and their littermate controls after CL treatment. β-actin was used as a loading control. (G) qRT-PCR analysis for BAT-selective genes in iWAT from AKO mice and their littermate controls after 1 week of the β3-adrenergic receptor agonist CL316,243 (1 mg/kg) injection. n = 3 biological replicates. (H) Oil red O staining of differentiated beige adipocytes (day 6) from WT and miR-22 KO mice. Scale bar: 100 μm (I) qRT-PCR analysis for BAT-selective genes and pan-adipocyte genes in differentiated beige adipocytes. n = 3 technical replicates. (J) Western blots for Ucp1 in differentiated beige adipocytes from WT and miR-22 KO mice. *$P < 0.05$, **$P < 0.01$, and ***$P < 0.001$ (two-tailed Student's *t*-test).

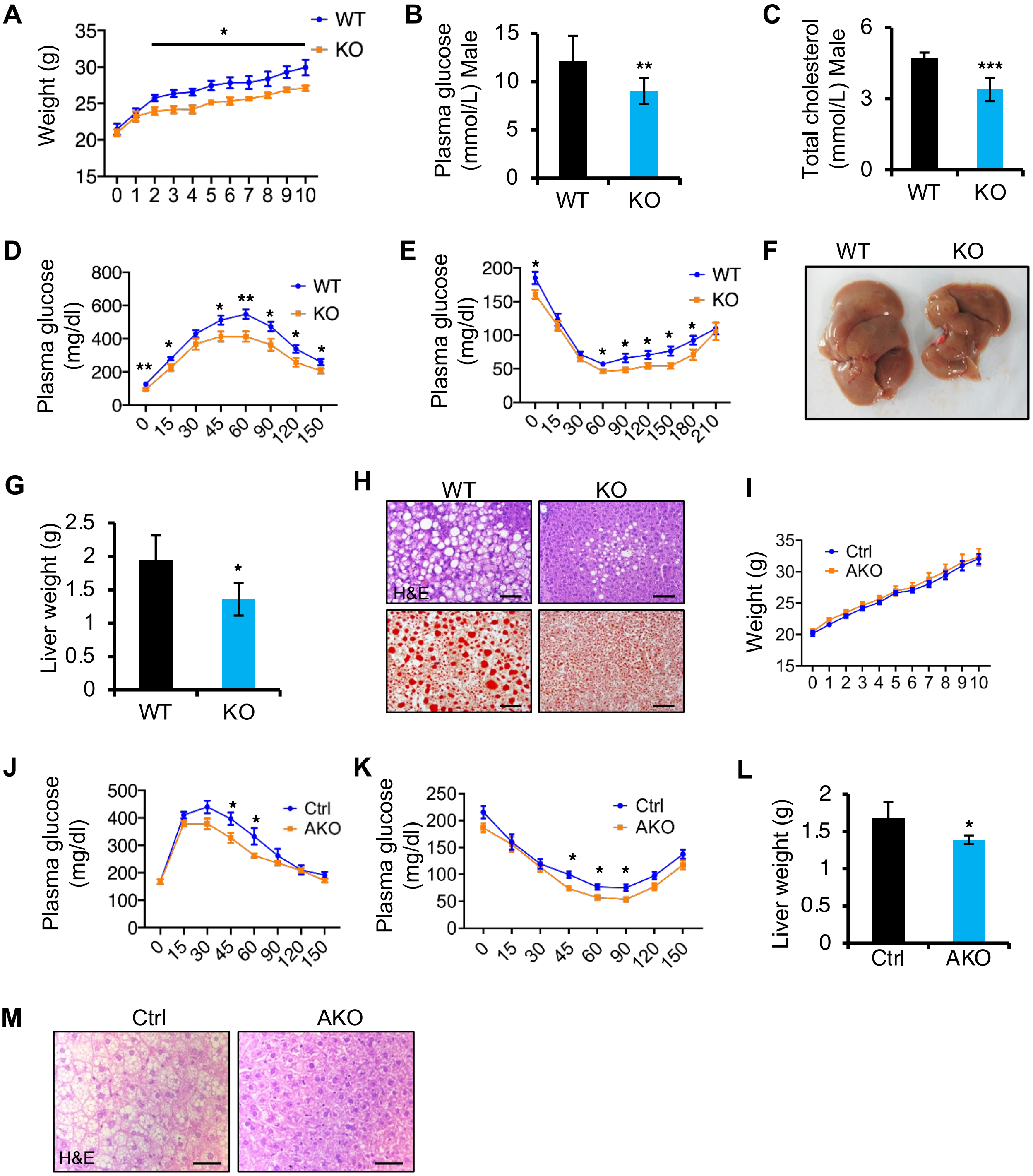
A
Weight (g)
35
30
25
20
15
0 1 2 3 4 5 6 7 8 9 10
*
WT
KO
B
Plasma glucose (mmol/L) Male
15
10
5
0
**
WT
KO
C
Total cholesterol (mmol/L) Male
6
3
0
***
WT
KO
D
Plasma glucose (mg/dl)
800
600
400
200
0
0 15 30 45 60 90 120 150
** * * ** * * *
WT
KO
E
Plasma glucose (mg/dl)
200
150
100
50
0
0 15 30 60 90 120 150 180 210
* * * * * *
WT
KO
F
WT
KO
G
Liver weight (g)
2.5
2
1.5
1
0.5
0
*
WT
KO
H
WT
KO
H&E
I
Weight (g)
35
30
25
20
15
0 1 2 3 4 5 6 7 8 9 10
Ctrl
AKO
J
Plasma glucose (mg/dl)
500
400
300
200
100
0
0 15 30 45 60 90 120 150
* *
Ctrl
AKO
K
Plasma glucose (mg/dl)
250
200
150
100
50
0
0 15 30 45 60 90 120 150
* * *
Ctrl
AKO
L
Liver weight (g)
2
1.5
1
0.5
0
*
Ctrl
AKO
M
Ctrl
AKO
H&E

Figure S6. miR-22 KO mice are protected against HFD-induced insulin resistance. (A) Quantification of body weights of WT (n = 10) and KO (n = 10) mice at HFD condition. (B-C) The levels of glucose (B) and total cholesterol (C) in serum from WT (n = 8) and KO (n = 8) male mice following 10 weeks of HFD under 16-hour fasted conditions. (D-E) The levels of glucose tolerance test (GTT) (D) and insulin tolerance test (ITT) (E) in WT (n = 8) and miR-22 KO (n = 8) mice following 10 weeks of HFD under 16-hour fasted conditions. (F) Macroscopic view of livers from WT (n = 4) and miR-22 KO (n = 4) male mice at the age of 18 weeks. The mice were treated with HFD for 10 weeks at age of 8 weeks. (G) Quantification of liver weights in panel F. (H) H&E and Oil Red O staining of livers from WT and miR-22 KO male mice in Panel F. Scale bar: 100 μm. (I) Quantification of body weights from control (n = 10) and miR-22 AKO (n = 10) male mice during HFD. (J-K) The levels of GTT (J) and ITT (K) in serum from control (n = 8) and miR-22 AKO (n = 8) mice following 10 weeks of HFD. (L) Liver weights of control and miR-22 AKO mice after 10 weeks of HFD (n = 5). The mice were treated with HFD for 10 weeks at age of 8 weeks. (M) H&E staining of liver from control and miR-22 AKO mice after 10 weeks of HFD (Scale bar: 50 μm). *$P < 0.05$, **$P < 0.01$, and ***$P < 0.001$ (two-tailed Student's *t*-test). Data are represented as the means ± SEMs (A, D, E, I, K, J) and others as the means ± SDs.

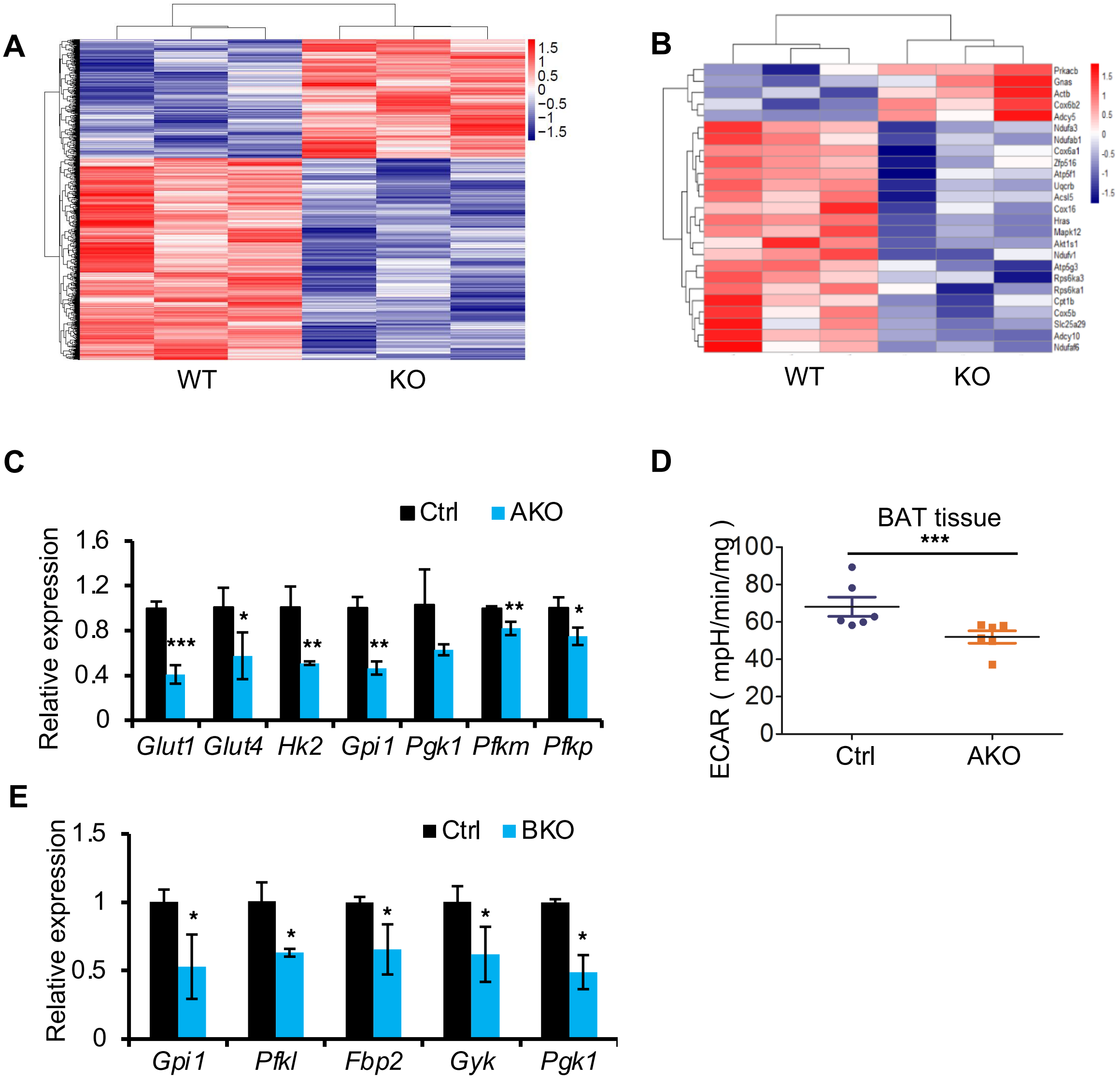

Figure S7. Glycolysis is suppressed in BAT from miR-22 conditional knockout mice. (A) Heatmaps of differentially expressed genes in BAT between WT and miR-22 KO mice. (B) Heatmaps of differentially expressed genes related to thermogenesis. (C) qRT-PCR analysis for the indicated glycolytic genes in BAT from control and miR-22 AKO mice (n = 3). (D) ECAR of BAT isolated from control and miR-22 AKO mice (n = 6). (E) qRT-PCR analysis for the indicated glycolytic genes in BAT from Ctrl and miR-22 BKO mice. n = 3 biological replicates. *$P < 0.05$, **$P < 0.01$, and ***$P < 0.001$ (two-tailed Student's *t*-test). Data are represented as the means ± SDs.

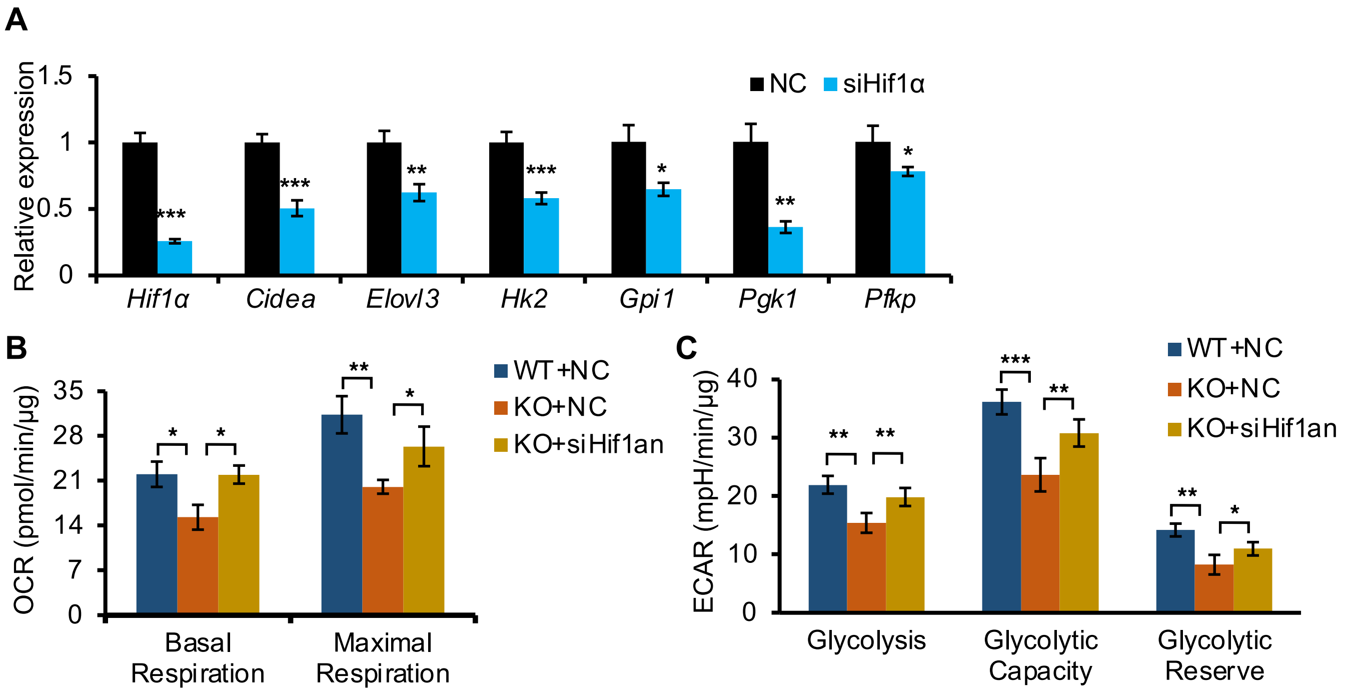

Figure S8. miR-22 promotes thermogenesis and glycolysis by directly suppressing Hif1an. (A) qRT-PCR analysis for the indicated genes in thermogenic and glycolytic pathways in differentiated primary brown adipocytes (day 6) upon Hif1α siRNA treatments. n = 4 technical replicates. (B-C) OCR (B) and ECAR (C) in differentiated miR-22 KO SVF cells at indicated conditions. *$P < 0.05$, **$P < 0.01$, and ***$P < 0.001$ (two-tailed Student's *t*-test). Data are represented as the mean ± SD.

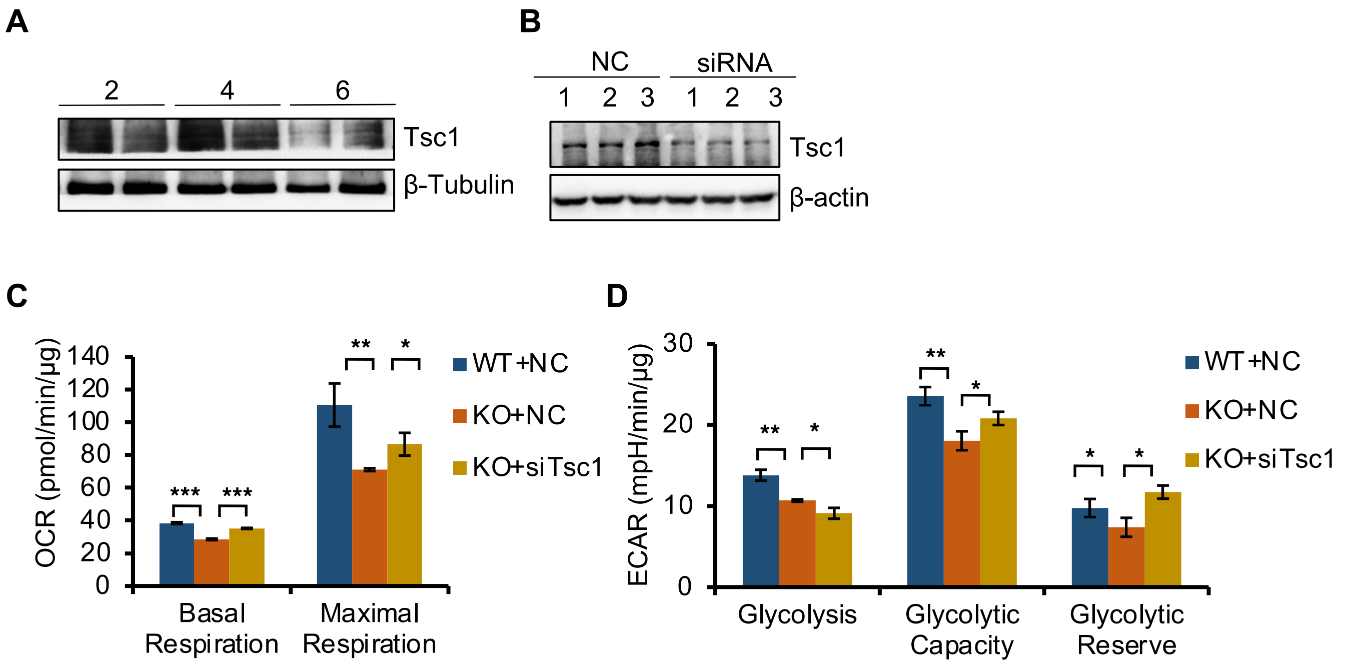

Figure S9. Identification of miR-22 direct targets in regulating mTORC1 signaling. (A) Western blots of Tsc1 during the differentiation of brown preadipocytes. β-Tubulin was used as a loading control. (B) Western blots of Tsc1 after siRNA treatment. β-actin was used as a loading control. (C-D) OCR (C) and ECAR (D) in differentiated primary brown adipocytes (day 6) at indicated conditions. *$P < 0.05$, **$P < 0.01$, and ***$P < 0.001$ (two-tailed Student's *t*-test). Data are represented as the mean ± SD.

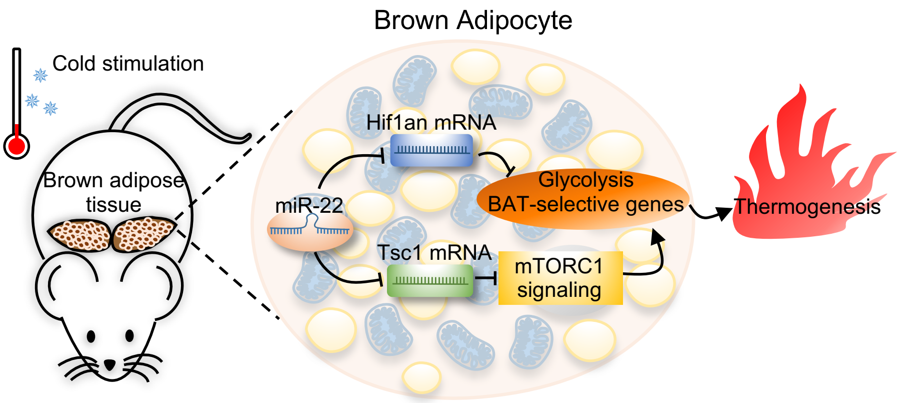

Figure S10. The working model of *miR-22* in regulating BAT thermogenesis.

Table S1. Primers used in the present study.

Gene	Forward	Reverse
Ucp1	CACCTTCCCGCTGGACACT	CCCTAGGACACCTTTATACCTAATGG
Prdm16	CAGCACGGTGAAGCCATTC	GCGTGCATCCGCTTGTG
Cidea	ATCACAACTGGCCTGGTTACG	TACTACCCGGTGTCCATTTCT
Pgc1α	CCCTGCCATTGTTAAGACC	TGCTGCTGTTCCTGTTTTC
Cox8b	GAACCATGAAGCCAACGACT	GCGAAGTTCACAGTGGTTCC
Cox5a	GCCGCTGTCTGTTCCATTC	GCATCAATGTCTGGCTTGTTGAA
C/EBPα	CAAGAACAGCAACGAGTACCG	GTCACTGGTCAACTCCAGCAC
Elovl3	TCCGCGTTCTCATGTAGGTCT	GGACCTGATGCAACCCTATGA
Adipoq	GCACTGGCAAGTTCTACTGCAA	GTAGGTGAAGAGAACGGCCTTGT
Fabp4	ACACCGAGATTTCCTTCAAACTG	CCATCTAGGGTTATGATGCTCTTCA
PPARγ	GTGCCAGTTTCGATCCGTAGA	GGCCAGCATCGTGTAGATGA
Hk2	TGATCGCCTGCTTATTCACGG	AACCGCCTAGAAATCTCCAGA
Gpi1	TCAAGCTGCGCGAACTTTTTG	GGTTCTTGGAGTAGTCCACCAG
Pfkl	GGAGGCGAGAACATCAAGCC	CGGCCTTCCCTCGTAGTGA
Pfkp	TGGTGCCATCATGCTATCTGA	GGTCGCACGTCTCGACAAT
Pfkm	TGTGGTCCGAGTTGGTATCTT	GCACTTCCAATCACTGTGCC
Fbp2	ACCCTGACCCGTTACGTTATG	ACATTCACGCTCCCCGAAATC
Gyk	TGAAGAAAGCGAAATCCGTTACT	CCCAAAGGCAGACTACAGAAG
Pgk1	ATGTCGCTTTCCAACAAGCTG	GCTCCATTGTCCAAGCAGAAT
Glut1	CAGTTCGGCTATAACACTGGTG	GCCCCCGACAGAGAAGATG
Glut4	GTGACTGGAACACTGGTCCTA	CCAGCCACGTTGCATTGTAG
Pkm1	CTGCTGTTTGAAGAGCTTGTG	GAGTCACGGCAATGATAGGA
Pkm2	TGCTGCAGTGGGGCCATTAT	GAGTCACGGCAATGATAGGA
Hif1an	GTCCCAGCTACGAAGTTACAGC	CAGTGCAGGATACACAAGGTTT
Hif1α	ACCTTCATCGGAAACTCCAAAG	CTGTTAGGCTGGGAAAAGTTAGG
Tsc1	ATGGCCCAGTTAGCCAACATT	CAGAATTGAGGGACTCCTTGAAG
Tbp	ACCCTTCACCAATGACTCCTATG	TGACTGCAGCAAATCGCTTGG
36B4	TTTGGGCATCACCACGAAAA	GGACACCCTCCAGAAAGCGA
	Primer sequences used for genotyping	
Mut-adipoq-cre	ACGGACAGAAGCATTTTCCA	GGATGTGCCATGTGAGTCTG
Ctrl-adipoq-cre	CTAGGCCACAGAATTGAAAGATCT	GTAGGTGGAAATTCTAGCATCATCC
miR-22$^{floxp/floxp}$	AGGGCCCCGGCTTTTACTGCTGAT	GGAGGGGAGGGAGGTATGGGTAGG
miR-22KO-mut	AGCTTGCCTGGGACTTAACC	
miR-22KO-wt	ACAGGAAAGCTGGGTGACAG	
miR-22KO-com	TGCATTTAGAAGCCTCTTGCT	

Table S2. siRNA or inhibitor used in the present study.

Gene	Forward	Reverse
miR-22 inhibitor	ACAGUUCUUCAACUGGCAGCUU	
miR-22 mimics	AAGCUGCCAGUUGAAGAACUGU	AGUUCUUCAACUGGCAGCUUUU
siHif1an	GGGAGGAAAUUAAAUUUCATT	UGAAAUUUAAUUUCCUCCCTT
siTsc1	GGAUGUACCCAUGUAACUUTT	AAGUUACAUGGGUACAUCCTT
siHif1α	GCUCACCAUCAGUUAUUUATT	UAAAUAACUGAUGGUGAGCTT

Table S3. The antibodies used in this study.

Antigen	Vendor	Catalog number
Ucp1	Abcam	ab10983
Prdm16	Abcam	ab106410
pS6	Cell Signaling Technology	4858
Hif1an	Abcam	ab187524
Hif1α	Abcam	ab16066
AKT	Cell Signaling Technology	9272
AKT-pS473	Cell Signaling Technology	4060
AKT-pT308	Cell Signaling Technology	13038
S6	Abcam	ab225676
4ebp1	Cell Signaling Technology	9644
p-p70 S6K	Cell Signaling Technology	9205
p70 S6K	Cell Signaling Technology	9202
p-4ebp1	Cell Signaling Technology	2855
Tsc1	Proteintech	20988-1-AP
β-actin	YEASEN	30101ES50
β-Tubulin	YEASEN	30301ES60
